# Differential miRNA plasma profiles associated with the spontaneous loss of HIV‐1 control: miR‐199a‐3p and its potential role as a biomarker for quick screening of elite controllers

**DOI:** 10.1002/ctm2.474

**Published:** 2021-07-04

**Authors:** Jenifer Masip, Carmen Gasca‐Capote, María Reyes Jimenez‐Leon, Joaquim Peraire, Alberto Perez‐Gomez, Verónica Alba, Ana‐Irene Malo, Lorna Leal, Carmen Rodríguez Martín, Norma Rallón, Consuelo Viladés, Montserrat Olona, Francesc Vidal, Ezequiel Ruiz‐Mateos, Anna Rull, ECRIS integrated in the Spanish AIDS Research Network (Annex S1)

**Affiliations:** ^1^ Universitat Rovira i Virgili Tarragona Spain; ^2^ Institut Investigació Sanitària Pere Virgili (IISPV) Tarragona Spain; ^3^ Hospital Universitari de Tarragona Joan XXIII Tarragona Spain; ^4^ Clinic Unit of Infectious Diseases Microbiology and Preventive Medicine, Institute of Biomedicine of Seville, IBiS Virgen del Rocío University Hospital/CSIC/University of Seville Seville Spain; ^5^ Vascular Medicine and Metabolism Unit, Research Unit on Lipids and Atherosclerosis Sant Joan University Hospital, Universitat Rovira i Virgili, IISPV Reus Spain; ^6^ Infectious Diseases Department ‐ HIV Unit, Hospital Clínic Barcelona, IDIBAPS University of Barcelona Barcelona Spain; ^7^ Centro Sanitario Sandoval, Hospital Clínico San Carlos Instituto de Investigación Sanitaria San Carlos (IdISSC) Madrid Spain; ^8^ HIV and Viral Hepatitis Research Laboratory Instituto de Investigación Sanitaria Fundación Jiménez Díaz, Universidad Autónoma de Madrid (IIS‐FJD, UAM) Madrid Spain; ^9^ Hospital Universitario Rey Juan Carlos Móstoles Madrid Spain

To the Editor:

People living with HIV (PLWH) who are able to maintain suppressed viral load (VL) for years in the absence of antiretroviral therapy (ART) are known as elite controllers (ECs). ECs represent a heterogeneous population in terms of virological, immunological, and clinical outcomes, and approximately 25% of ECs lose viral control overtime. The study of the mechanisms leading to the loss of viral control in ECs is crucial for the identification of differential markers for the design of novel eradication and immunotherapeutic strategies.

Previously, we identified virological and immunological factors involved in the spontaneous loss of viral control,^1^ and we also demonstrated that proteomics and metabolomics are powerful tools to identify potential biomarkers and therapeutic targets in ECs.^2^, ^3^ Additionally, genome‐wide associations and transcriptome analyses have also been described for ECs and compared to other phenotypes of PLWH,^4^, ^5^ in particular the study of specific microRNA (miRNA) expression profiles.^5^ miRNAs play vital roles in development, apoptosis, and oncogenesis by interfering with gene expression at the post‐transcriptional level.^6^ In HIV/AIDS scenario, the most relevant feature is that some miRNAs can modulate HIV replication by directly targeting HIV RNA or targeting messenger RNA (mRNA) of cell factors necessary for HIV replication.^7^

Our study conducted in 18 ECs (Figure S1, Table S1), 12 individuals who experienced a loss of spontaneous viral HIV‐1 control (transient controllers, TCs) and six ECs who persistently maintained viral control during the same follow‐up period (persistent controllers, PCs), showed an upregulated plasma miRNA profile in TCs before and after the loss of viral control.

First, 23 miRNAs were found differentially expressed in TCs compared to PCs at the preloss time point (Figure 1A, Table S2). From the 23 miRNAs, 22 miRNAs were positively correlated with viral load (VL), seven miRNAs were positively correlated with CD4^+^ T‐cell counts, and 11 miRNAs were positively correlated with CD8^+^ T‐cell counts (Table S3). Interestingly, the spontaneous loss of viral control in ECs can be defined by the expression of hsa‐miR‐27a‐3p, hsa‐miR‐376a‐3p, and hsa‐miR‐199a‐3p (Figure 1B), as confirmed by the diagnostic accuracy determined by the ROC analysis (Figure 1C). Notably, hsa‐miR‐27a‐3p, hsa‐miR‐376a‐3p, and hsa‐miR‐199a are tightly connected and related to lipid metabolism (Figure 1D). Then, we also evaluated the plasma miRNA profile in TCs under the postloss condition and we found 38 miRNAs differentially expressed among groups, suggesting that viremia strongly influences the plasma miRNA profile of ECs (Figure 2A, Table S4). And again, these significantly expressed miRNAs among groups were also related to relevant genes linked to lipid pathways (Figure 2B), and some of them positively correlated with VL, CD4^+^ T‐cell counts and CD8^+^ T‐cell counts (Table S5). Moreover, of the 23 miRNAs significantly differentially expressed under the preloss condition and the 38 miRNAs significantly differentially expressed under the postloss condition, only the upregulation of 19 miRNAs overlapped (Figure 3A). Noteworthy, the expression of hsa‐miR‐199a‐3p showed an optimal percentage of separation and an ability to differentiate between both groups of ECs before and after the loss of viral control (Figure 3B). Accordingly, the upregulation of hsa‐miR‐199a‐3p could be related to lipid dysregulation in TCs, which in turn may potentiate the activation of a cytokine deregulation^1^ and in last term bias the virological control in TCs.

**FIGURE 1:**
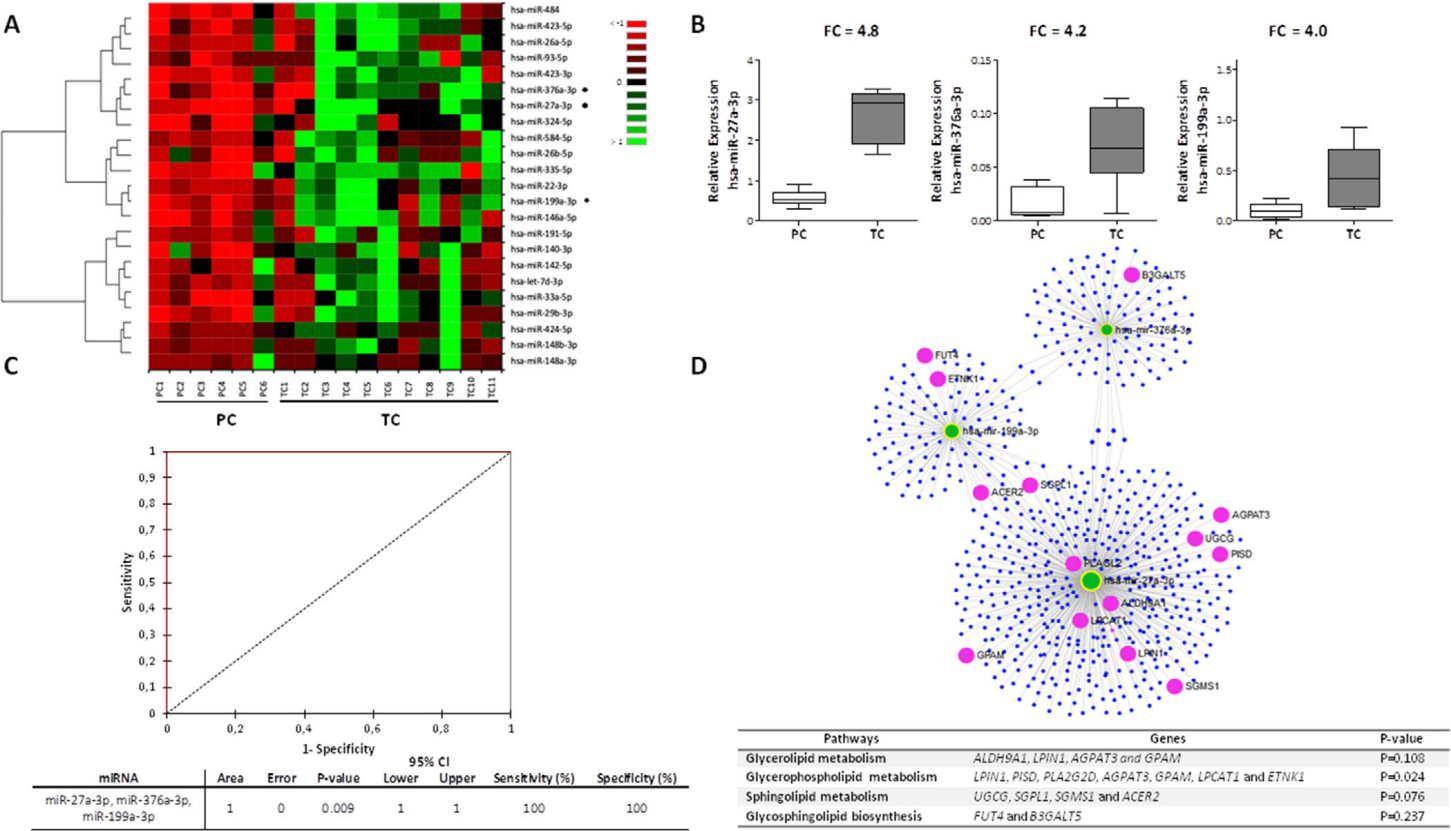
miRNA profile distinguishes TCs and PCs under the preloss condition. (A) Hierarchical clustering heatmap of a set of the 23 significantly expressed miRNAs; hierarchical combined tree shows the clusterization of miRNAs. For PCs, only one measurement was assessed during the follow‐up and used as the control group for comparison to preloss and postloss time points in TCs. For TCs (*n* = 11), one patient was excluded for lack of expression data values in the preloss condition. Indicated with black dots, next to the miRNAs list, the highly upregulated miRNAs (FC > 4). Columns represent each patient, while rows correspond to significant miRNAs. (B) Plasma miRNA profile in PLWH‐ECs. hsa‐miR‐27a‐3, hsa‐miR‐376a‐3p, and hsa‐miR‐199a‐3p were the most highly upregulated miRNAs in TCs before the loss of viral control compared to PCs. Statistical analysis was carried out by a nonparametric Mann–Whitney *t*‐test. Data are represented as box and whiskers (min to max values). (C) Receiver operating characteristic curves (ROC curves) from the combination of hsa‐miR‐27a‐3p, hsa‐miR‐199a‐3p, and hsa‐miR‐376a‐3p (FC > 4) in TCs under the preloss condition (AUC = 1). (D) Network analysis display of the miRTarBase v8.0 database showing the association of the significantly upregulated miRNAs and their target genes in TCs compared to PCs before the loss of viral control. Cluster hubs shown in green circles indicate miRNAs, whereas blue circles depict their target genes (in purple circles the highlighted genes involved in the lipid metabolism as commented in the text). Bottom table displays some of the genes involved with these miRNAs that link hsa‐miR‐27a‐3p and hsa‐miR‐199a‐3p

**FIGURE 2:**
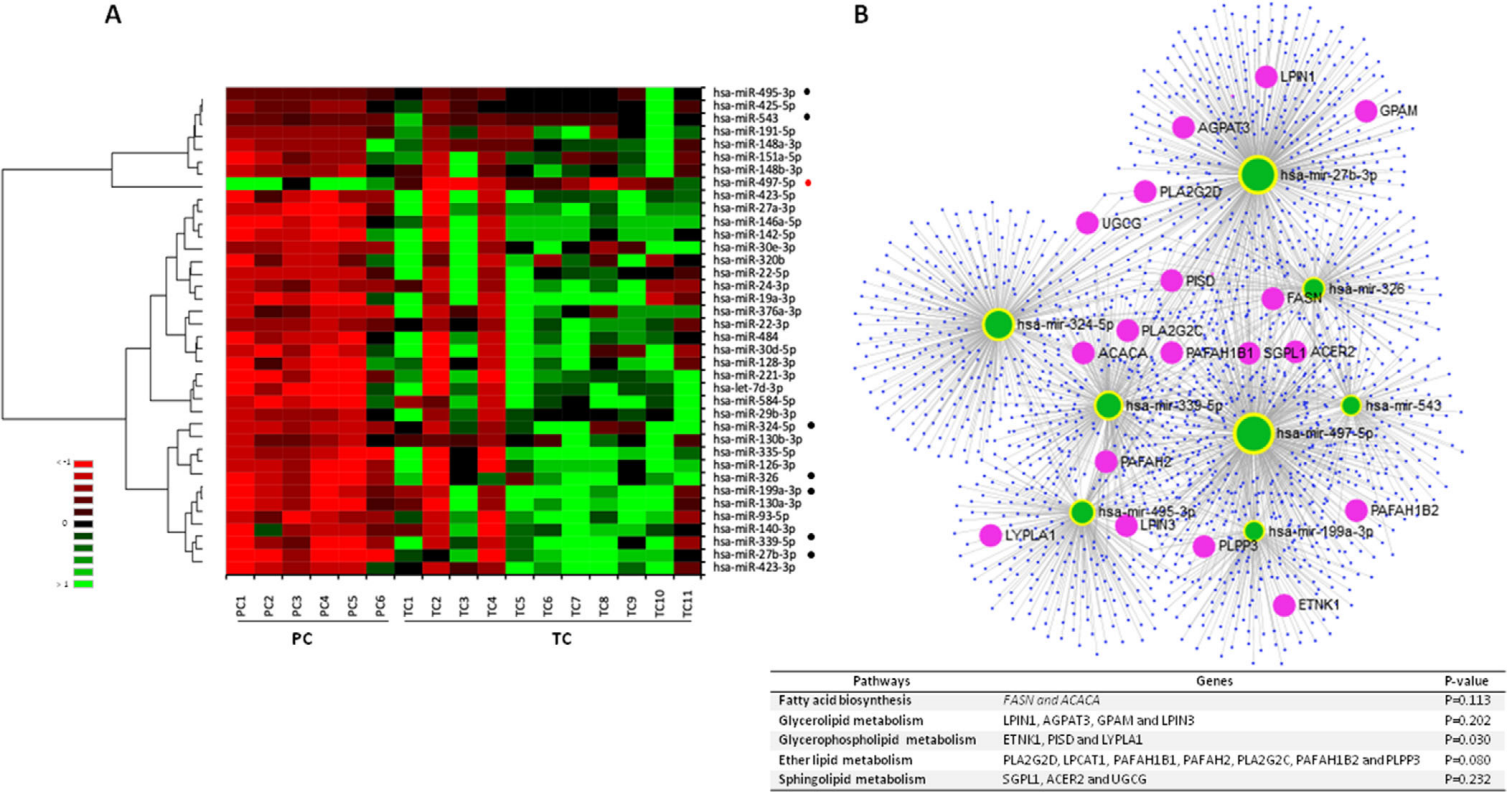
miRNA profile distinguishes TCs and PCs under the postloss condition. (A) Hierarchical clustering heatmap of the set of 38 significantly expressed miRNAs; hierarchical combined tree shows the clusterization of the miRNAs. For PCs, only one measurement was assessed during the follow‐up and used as the control group for comparison to preloss and postloss time points in TCs. For TCs (*n* = 11), one patient was excluded for lack of expression data values in the preloss condition. Indicated with black dots, next to the miRNAs list, the highly upregulated miRNAs and in red dots the downexpressed miRNA in TCs under the postloss condition. Columns represent each patient, while rows correspond to significant miRNAs. (B) Network display of the miRTarBase v8.0 database showing the association of the significantly upregulated miRNAs and their target genes in TCs compared to PCs after the loss of viral control. Cluster hubs shown in green circles indicate miRNAs, whereas blue circles depict their target genes (in purple circles are the highlighted genes involved in the lipid metabolism as commented in the text). Bottom table displays some of KEGG pathways related with these seven most highly upregulated miRNAs under the postloss condition

**FIGURE 3:**
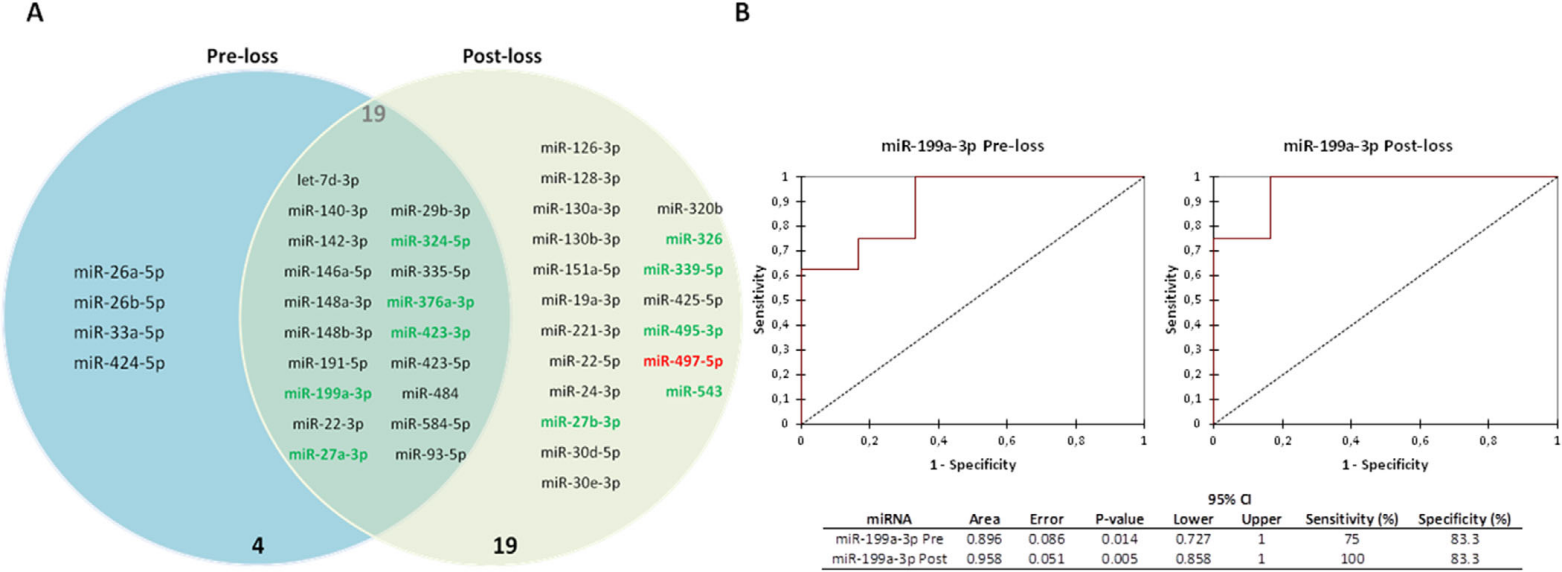
miRNA profile in TCs under the preloss condition differs from the postloss condition. (A) Venn's diagram representation showing the overlapping of the significantly expressed miRNAs in TCs, from both conditions, compared to PCs. In green are the highly expressed miRNAs; in red the downregulated miRNAs. (B) Logistic regression and receiver operating characteristic curves (ROC curves) from preloss and postloss relative expression elucidated hsa‐miR‐199a‐3p as the most reliable potential biomarker for the prediction of the spontaneous loss of viral control in ECs

Thus, our results confirmed differences in the expression of some miRNAs with target sites located in viral RNA regions encoding viral accessory proteins, suggesting the emerging concept that upregulated host‐derived miRNAs in TCs might act as antiviral defence mechanism directly affecting important steps during HIV infection and also playing a key role in HIV immune pathogenesis. Notably, hsa‐miR‐423‐3p targets the *gag* gene, hsa‐miR‐29a/29b and hsa‐miR‐326 target the *nef* gene, and hsa‐miR‐324‐5p targets the *vif* gene^6^, ^8^ (Figure 4).

**FIGURE 4:**
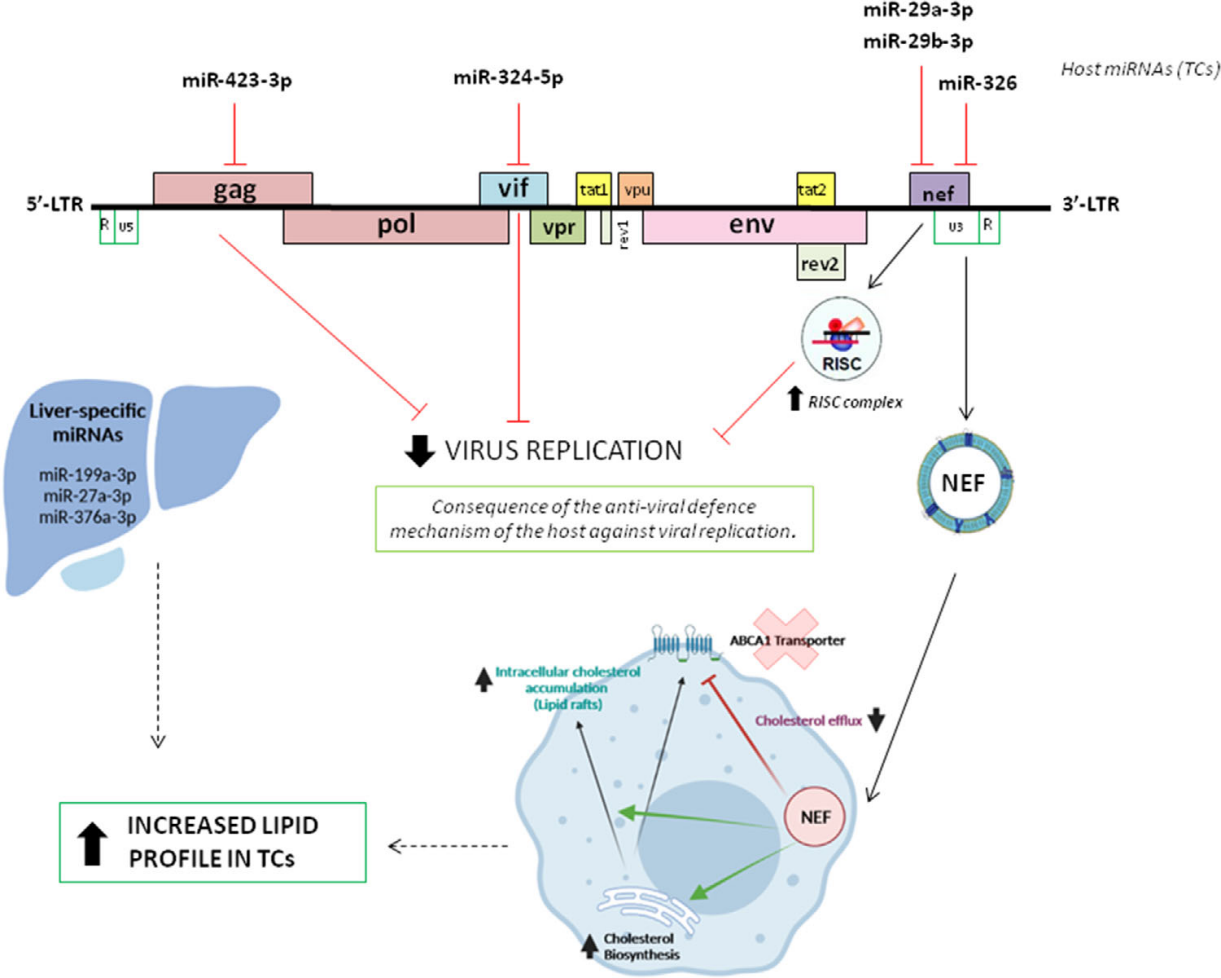
Proposed mechanisms of effect of the differential miRNA profile associated with the spontaneous loss of HIV‐1 control. Host miRNAs that have been experimentally shown to regulate HIV expression are illustrated with the target sites located in viral RNA regions encoding important viral accessory proteins. The inhibitory effect of HIV replication by hsa‐miR‐423‐3p (*gag* gene), hsa‐miR‐324‐5p (*vif* gene), hsa‐miR‐29a/29b miRNA, and hsa‐miR‐326 (both target the *nef* gene) could be a mechanism against the virus after losing viral control in TCs. Of note, hsa‐miR‐29a/b can bind directly to the HIV‐1 mRNA (*nef* gene) increasing its association with proteins in the RISC complex, which leads to the inhibition of the translation of viral proteins and viral replication. Moreover, during HIV infection, the viral accessory protein Nef (via exosomes) impairs activity of cholesterol transporter ABCA1, inhibiting cholesterol efflux and also increases the biosynthesis and accumulation of cholesterol from macrophages. On the other hand, the liver‐specific miRNAs hsa‐miR‐miR‐199a‐3p, hsa‐miR‐miR‐27a‐3p, and hsa‐miR‐376a‐3p were strongly related to lipid and lipoprotein metabolism (Figure 1D). Concretely, hsa‐miR‐27 is one of the key regulators of the expression of ABCA1 and LDL receptor, whereas hsa‐miR‐199a‐3p is implicated in fatty acid and cholesterol biosynthesis. These results could be associated to the increased lipid profile in TCs after the loss of viral control. In red are represented the inhibited actions and in black are represented the indirect actions

One mechanism associated to the inhibitory effect of HIV replication could be the interaction of *nef* with the RISC complex, which leads to the inhibition of the translation of viral proteins and viral replication. On the other hand, Nef‐containing exosomes, taken up by macrophages, have been suggested to impair lipid cholesterol efflux, causing intracellular cholesterol accumulation, which consequently affects the risk of cardiovascular diseases in PLWH.^9^, ^10^ Several miRNAs can regulate different steps of HDL‐C metabolism, and recent studies have promoted the importance of miRNAs in controlling LDL metabolism and in the regulation of genes involved in very low‐density lipoprotein (VLDL) secretion. Our results are in accord with these data since the LDL metabolism resulted to be increased in TCs under the postloss condition (Figure S2 and Figure S3).^2^ Additionally, some of the most representative host‐derived miRNAs associated with the spontaneous loss of viral control overtime are liver‐specific miRNAs, being implicated in fatty acid and cholesterol biosynthesis. Thus, our results suggest that disturbance in lipoprotein levels, mostly induced by upregulation of some liver‐specific miRNAs, may be highly associated with the immunological factors behind the loss of viral control in TCs. It is known that HIV infection is characterized by a high energy demand to reprogram the cells to aerobic glycolytic pathways that consequently may increase anabolic metabolism.^2^ So, the increased lipid profile in TCs before the loss of viral control could be a consequence of the antiviral defence mechanism of the host against viral replication.

The main limitation of this work is the small sample size. However, it must be highlighted that these patients are rare, and it is difficult to have a follow‐up with sequentially stored samples. Despite the relatively low number of patients, we were able to have a tight follow‐up of TCs with samples before and after the loss of virological control. Validation studies are needed to establish the proposed miRNAs as biomarkers for the loss of viral control in ECs.

In conclusion, our study reveals a specific host‐derived miRNA pattern in ECs that may be used as a biomarker for quick screening of the virological and immunological progression in ECs, and we confirmed that viremia induces increased LDL metabolism in TCs. Notably, the expression of liver‐specific hsa‐miR‐199a‐3p showed an optimal percentage of separation and an ability to differentiate between both groups of ECs before and after the loss of viral control.

## ETHICS APPROVAL AND CONSENT TO PARTICIPATE

All research protocols were approved and carried out according to the recommendations of the Ethical Committee for Clinical Research following the rules of Good Clinical Practice from the Institut d'Investigació Sanitària Pere Virgili (CEIm IISPV). The CEIM IISPV is an independent committee, made up of health and non‐health professionals, which supervises the correct compliance of the ethical principles governing clinical trials and research projects that are carried out in our region, specifically in terms of methodology, ethics, and laws. All participants gave written informed consent in agreement with the Declaration of Helsinki.

## FUNDING INFORMATION

Fondo de Investigación Sanitaria‐ISCIII‐FEDER: PI13/0796, PI16/00503, PI16/0684, PI18/1532, PI19/01127, PI19/01337, PI20/00326, RD16/0025/0006, RD16/0025/0020, INT20/00031, FI19/00083, FI17/00186, CP19/00146, CPII19/00025. European Regional Development Fund/European Social Fund; Programa de Suport als Grups de Recerca AGAUR, Grant Number: 2017SGR948; Gilead Fellowship Program, Grant Number: GLD14/293; SPANISH AIDS Research Network, Grant Numbers: RD16/0025/0006, RD16/0025/0020 (ISCIII‐FEDER, Spain). Programa de Intensificación de Investigadores, Grant Number: INT20/00031; Consejo Superior de Investigaciones Cientificas (CSIC); ISCIII‐FEDER, PFIS programme, Grant Numbers: FI19/00083, FI17/00186; IISPV: 2019/IISPV/05 (Boosting Young Talent); GeSIDA “III Premio para Jóvenes Investigadores 2019″; Instituto de Salud Carlos III (ISCIII), Grant Number: CP19/00146; Universitat Rovira i Virgili, Grant Number: 2019PMF‐PIPF‐18; Instituto de Salud Carlos III (ISCIII), Grant Number: CPII19/00025

## AUTHOR CONTRIBUTIONS

All authors reviewed and approved the submitted version of the manuscript. Experimental design: Jenifer Masip, Carmen Gasca‐Capote, Ezequiel Ruiz‐Mateos, and Anna Rull. Intellectual guidance: Consuelo Viladés, Joaquim Peraire, Francesc Vidal, Anna Rull, and Ezequiel Ruiz‐Mateos. Recruitment of participants: Jenifer Masip, Ana‐Irene Malo, Lorna Leal, Carmen Rodríguez Martín, Norma Rallón, Consuelo Viladés, Joaquim Peraire, Montserrat Olona, and Francesc Vidal. Sample procurement: Jenifer Masip and Verónica Alba. Data collection: Ezequiel Ruiz‐Mateos, Ana‐Irene Malo, Lorna Leal, Carmen Rodríguez Martín, Norma Rallón, Consuelo Viladés, Joaquim Peraire, and Montserrat Olona. Data analysis and interpretation: Jenifer Masip, María del Carmen Gasca‐Capote, María Reyes Jimenez‐Leon, Alberto Perez‐Gomez, Ezequiel Ruiz‐Mateos, and Anna Rull. Manuscript preparation: Jenifer Masip, María del Carmen Gasca‐Capote, Ezequiel Ruiz‐Mateos, and Anna Rull. Study design, data analysis, and article development: Jenifer Masip, Anna Rull, Ezequiel Ruiz‐Mateos, and Francesc Vidal. Reviewed and edited the manuscript: Francesc Vidal, Anna Rull, and Ezequiel Ruiz‐Mateos.

## DATA AVAILABILITY STATEMENT

The data that support the findings of this study are available from the corresponding author upon reasonable request.

## Supporting information

SUPPORTING INFORMATION

## References

[1] Pernas M , Tarancón‐Diez L , Rodríguez‐Gallego E , et al. Factors leading to the loss of natural elite control of HIV‐1 infection. J Virol. 2018;92(5):e01805‐e01817. 29212942 10.1128/JVI.01805-17 PMC5809746

[2] Tarancon‐Diez L , Rodríguez‐Gallego E , Rull A , et al. Immunometabolism is a key factor for the persistent spontaneous elite control of HIV‐1 infection. EBioMedicine. 2019;42:86‐96. 30879922 10.1016/j.ebiom.2019.03.004 PMC6491381

[3] Rodríguez‐Gallego E , Tarancón‐Diez L , Garciá F , et al. Proteomic profile associated with loss of spontaneous human immunodeficiency virus type 1 elite control. J Infect Dis. 2019;219(6):867‐876. 30312441 10.1093/infdis/jiy599

[4] Egaña‐Gorroño L , Escribà T , Boulanger N , et al. Differential MicroRNA expression profile between stimulated PBMCs from HIV‐1 infected elite controllers and viremic progressors. PLoS One. 2014;9(9):e106360. 25225963 10.1371/journal.pone.0106360 PMC4165582

[5] Rotger M , Dang KK , Fellay J , et al. Genome‐wide mRNA expression correlates of viral control in CD4+ T‐cells from HIV‐1‐infected individuals. PLoS Pathog. 2010;6(2):e1000781. 20195503 10.1371/journal.ppat.1000781 PMC2829051

[6] Hariharan M , Scaria V , Pillai B , Brahmachari SK . Targets for human encoded microRNAs in HIV genes. Biochem Biophys Res Commun. 2005;337(4):1214‐1218. 16236258 10.1016/j.bbrc.2005.09.183

[7] Klase Z , Houzet L , Jeang KT . MicroRNAs and HIV‐1: complex interactions. J Biol Chem. 2012;287(49):40884‐40890. 23043098 10.1074/jbc.R112.415448 PMC3510792

[8] Ahluwalia JK , Khan SZ , Soni K , et al. Human cellular microRNA hsa‐miR‐29a interferes with viral nef protein expression and HIV‐1 replication. Retrovirology. 2008;5:117. 19102781 10.1186/1742-4690-5-117 PMC2635386

[9] Aryal B , Singh AK , Rotllan N , Price N , Fernández‐Hernando C . MicroRNAs and lipid metabolism. Curr Opin Lipidol. 2017;28(3):273‐280. 28333713 10.1097/MOL.0000000000000420 PMC5667558

[10] Mujawar Z , Rose H , Morrow MP , et al. Human immunodeficiency virus impairs reverse cholesterol transport from macrophages. PLoS Biol. 2006;4(11):1970‐1983. 10.1371/journal.pbio.0040365 PMC1629034 17076584

