# Supplementary material for: Differential miRNA plasma profiles associated with the spontaneous loss of HIV‐1 control: miR‐199a‐3p and its potential role as a biomarker for quick screening of elite controllers
Source: Clin Transl Med. 2021 Jul 4;11(7):e474. doi: 10.1002/ctm2.474 (PMC8255061; doi:10.1002/ctm2.474)
Supplement: Supplementary file 1 — SUPPORTING INFORMATION [file CTM2-11-e474-s001.pdf]

Supporting information

Differential miRNA plasma profiles associated with the spontaneous loss of HIV-1 control: miR-199a-3p and its potential role as a biomarker for quick screening of elite controllers

Letter-to-Editor with previous submission number CTM2-2021-04-0689

Running title: miR-199a-3p distinguishes ECs phenotypic profiles

Jenifer Masip[1,2,3#], María del Carmen Gasca-Capote[4#], María Reyes Jimenez-Leon[4], Joaquim Peraire[1,2,3], Alberto Perez-Gomez[4], Verónica Alba[1,2,3], Ana-Irene Malo[5], Lorna Leal[6], Carmen Rodríguez Martín[7], Norma Rallón[8], Consuelo Viladés[1,2,3], Montserrat Olona[1,2,3], Francesc Vidal[1,2,3], Ezequiel Ruiz-Mateos[4*], Anna Rull[1,2,3*] ECRIS integrated in the Spanish AIDS Research Network (Annex S1).

[1]Universitat Rovira i Virgili, Tarragona, Spain

[2]Institut Investigació Sanitària Pere Virgili (IISPV), Tarragona, Spain

[3]Hospital Universitari de Tarragona Joan XXIII, Tarragona, Spain

[4]Clinic Unit of Infectious Diseases, Microbiology and Preventive Medicine, Institute of Biomedicine of Seville, IBiS, Virgen del Rocío University Hospital/CSIC/University of Seville, Seville, Spain.

[5]Vascular Medicine and Metabolism Unit, Research Unit on Lipids and Atherosclerosis, Sant Joan University Hospital, Universitat Rovira i Virgili, IISPV, Reus, Spain.

[6]Infectious Diseases Department – HIV Unit, Hospital Clínic Barcelona, IDIBAPS, University of Barcelona, Spain.

[7]Centro Sanitario Sandoval, Hospital Clínico San Carlos, Instituto de Investigación Sanitaria San Carlos (IdISSC), Madrid, Spain.

[8]HIV and Viral Hepatitis Research Laboratory, Instituto de Investigación Sanitaria Fundación Jiménez Díaz, Universidad Autónoma de Madrid (IIS-FJD, UAM), Madrid, Spain. Hospital Universitario Rey Juan Carlos, Móstoles, Spain.

# These authors contributed equally to this paper and should be considered as primary co-authors.

* These authors contributed equally to this paper, and should both be considered as senior co-authors.

Correspondence:

Francesc Vidal Marsal
Department of Internal Medicine and Infectious Diseases,
Hospital Universitari de Tarragona Joan XXIII, IISPV, Universitat Rovira i Virgili
c/Mallafré Guasch, 4; Tarragona, Spain 43007
Phone: +34977295800 ext. 1963; FAX: +34977295833
fvidalmarsal.hj23.ics@gencat.cat

People living with HIV (PLWH) who are able to maintain suppressed viral load (VL) for years in the absence of antiretroviral therapy (ART) are known as elite controllers (ECs). However, ECs constitute a heterogeneous subset of PLWH in terms of virological, immunological and clinical outcomes. Some ECs attain very low $CD4^+$ T cell counts, a minority of ECs show clinical progression to AIDS-associated pathologies, and a group more frequently develops non-AIDS-defining events (nADEs) associated with high levels of persistent inflammation markers [1,2]. Regarding viral progression, some individuals control the virus for a long time (persistent elite controllers, PCs), while others, approximately 25% of ECs, lose their viral control over time, a subset of EC known as transient controllers (TC) [3].

Multiple studies have attempted to establish the specific mechanisms involved in the spontaneous loss of control in ECs, from virus factors associated with losing immunological and viral control to host factors that increase the probability of immunological progression [4–6], suggesting that pathogenic mechanisms related to HIV infection are still active in some ECs.

miRNAs have been discovered in many fields because of their interest in the identification of specific profiles associated with viral infections [7–9]. miRNAs, among the most important types of RNAis that can target multiple gene transcripts, are small noncoding oligoribonucleotide molecules with a single-stranded structure of 19–25 nt created by RNA polymerase II (Pol II) transcripts [10,11]. Specific miRNA patterns between ECs and viral progressors (VPs) [7] and long-term non-progressors (LTNPs) have been reported, elucidating the potential roles of miRNAs and their importance in viral replication and disease progression [12]. Interestingly, some evidence also suggests that HIV infection may affect lipid metabolism. Differences in miRNAs released into the bloodstream between HIV-positive and HIV-negative groups revealed miRNA profiling associated with the development of neurocognitive disorders, atherosclerosis and metabolic dysfunction [13,14].

Thus, the aim of this study was to identify the mechanism that may lead to a spontaneous loss of viral control at the gene transcription level. In our previous studies, we observed changes in proteins and metabolites in ECs even before they lost viral control. We suggest that the miRNA pattern may distinguish TCs from PCs before the spontaneous loss of viral control and that the expression pattern may be related to immunological and clinical outcomes.

MATERIALS AND METHODS

Patients and study design

ECs were defined as individuals with undetectable VL (viral load) (<50 HIV-1-RNA copies/ml) in the absence of ART for at least 12 months of follow-up. A total of 18 ECs were included in the study based on frozen plasma samples available in the Spanish HIV HGM BioBank belonging to the AIDS Research Network and with data registered in the RIS cohort of HIV Controllers Study Group (ECRIS) based on the study design[4] (Figure S1A). From the 18 ECs analysed, 12 individuals who experienced a loss of spontaneous viral HIV-1 control (2 measurements of VL above the detection limit in 12 months) were classified as transient controllers (TCs), and another group of 6 ECs who persistently maintained viral control during the same follow-up period were called persistent controllers (PCs). The Biobanc IISPV (B.0000853 + B.0000854)

integrated into the Biobanks and Biomodels Platform (PT20/00197) has supported the present study.

For TCs, measurements in two different follow-up time points were assessed, one determination before the loss of viral control as the pre-loss condition (one measurement during the previous 24 months of losing viral control) and another measurement in the closest follow up time point after the loss of viral control, post-loss condition (one measurement along the 12 months of follow-up after the viral rebound). For PCs, only one measurement was assessed during the follow-up and used as the control group for comparison to the pre-loss and post-loss time points in TCs (Figure S1B), after checking that no changes were found during the follow-up.

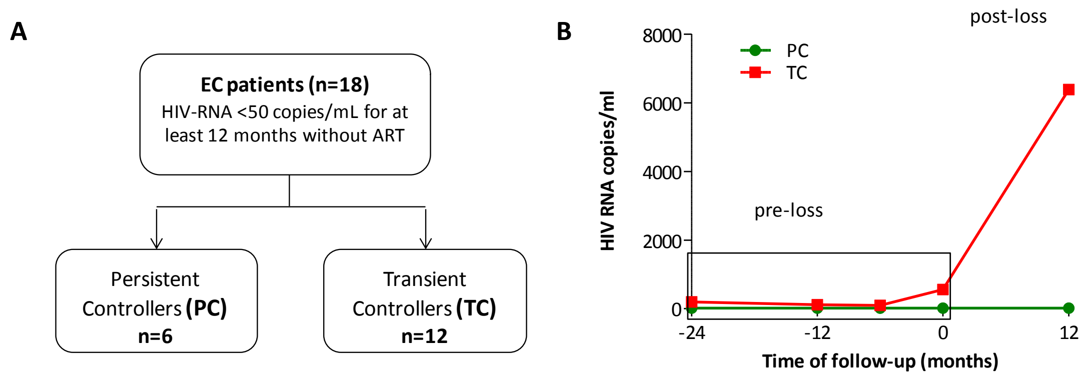

Figure S1. Flow chart and schematic representation illustrating subject cohort enrolment and study design. **A)** A total of 18 HIV-ECs with VL determinations below the detection limit (<50 HIV-1-RNA copies/ml) in the absence of ART for at least 12 months were included in the study and classified into two groups according to their viral control in transient controllers (TCs, n=12) and persistent controllers (PCs, n=6). **B)** Follow-up time points, for TCs two determinations were assessed, one determination before the loss of viral control ("pre-loss", from T-24 to T-0) and another one after the loss of viral control ("post-loss", from T0 to T+12) compared to one determination in PCs.

RNA isolation and quantification

Total RNA was isolated from frozen plasma samples according to the manufacturer's instructions using the MagMAX mirVana total RNA isolation kit (Applied Biosystems, Thermo Fisher Scientific's). RNA concentration was calculated using the µDrop plate (Thermo Fisher) and a Varioskan LUX Multimode Microplate Reader with the SkanIt Software. The absorbance was read at 260nm and 280nm (260nm/280nm RNA/protein). The RNA concentration (µg/µL) in each sample was calculated as Abs 260nm * dilution factor (500) * 40.

miRNA expression profiling using Taqman low-density arrays (TLDAs)

RNA (1-10ng) of the sample isolation procedure was reverse transcribed in a universal reverse transcription (RT) chemistry using TaqMan Advanced miRNA cDNA Synthesis Kit (Applied Biosystems, Thermo Fisher Scientific's) to prepare the cDNA template. miRNA expression profiles were obtained using TaqMan™ Advanced miRNA Human Serum/Plasma Card v22 (A34717, Applied Biosystems, Thermo Fisher Scientific's), containing 384-well microfluidic card format (hsa-miR). Subsequently, 220µL of cDNA (miR-Amp reaction product) was diluted and assayed (1:10) in RNase-free water and 440µL of TaqMan Fast Advanced Master Mix (2X) per card. One card for profiling of up to 188 unique miRNAs in one serum/plasma sample,

including endogenous and exogenous miRNA controls for normalization of data results. Real-time PCR reactions were performed using the 7900HT Fast Real-time PCR system. Thermal-cycling conditions according to the protocol were enzyme activation at 92˚C for 10 minutes by 1 cycle, denature phase at 95°C for 1 second and anneal/extend at 60°C by 40 cycles.

Liposcale test: NMR lipoprotein measurements

The Liposcale Test was performed in the Biosfer Teslab (Reus, Spain) for lipoprotein analysis [15]. All 1HNMRspectra were recorded at 310 K on a Bruker Avance III 600 spectrometer operating at a proton frequency of 600·20 MHz. Lipid concentrations (i.e., triglycerides and cholesterol), lipoprotein sizes and particle numbers for very-low-density lipoprotein (VLDL) (38.6–81.9 nm), low-density lipoprotein (LDL) (18.9–26.5 nm) and high-density lipoprotein (HDL) (7.8–11.5 nm) classes, as well as the particle numbers of nine subclasses, namely, large, medium, and small VLDL, LDL and HDL (a total set of 26 variables), were measured in 2D spectra from diffusion-ordered NMR spectroscopy (DOSY) experiments using the Liposcale test [15]. Briefly, the cholesterol and triglyceride concentrations of the main lipoprotein fractions were predicted using partial least squares (PLS) regression models. Then, the methyl proton resonances of the lipids in lipoprotein particles were decomposed into nine Lorentzian functions representing nine lipoprotein subclasses and the mean particle size of every main fraction was derived by averaging the NMR area of each fraction by its associated size. Finally, we calculated the particle numbers of each lipoprotein main fraction by dividing the lipid volume by the particle volume of a given class and we used the relative areas of the lipoprotein components used to decompose the NMR spectra to derive the particle numbers of the nine lipoprotein subclasses.

Statistical analysis

Categorical data were compared through a chi-squared test; whereas continuous data were compared using the non-parametric Mann-Whitney U test. Correlations between variables were assessed using the Spearman test. Differences between paired samples were determined using the non-parametric Wilcoxon test.

Taqman low-density arrays (TLDAs) were run in the 7900HT Fast Real-time PCR system and the signals were read with SDS 2.3 Software (Applied Biosystems, Foster City, CA, USA) and data normalization and interpretation were done by the ΔΔCT method. Raw CT (cycle threshold: the number of cycles required for the fluorescent signal to cross the threshold) values of the expression of each miRNA were obtained using automatic thresholding of all the processed data together with the Expression Suite Software v1.3 (Applied Biosystems, Thermo Fisher Scientific's). These miRNAs with Ct values >37 and not amplified were omitted from the analysis. For each sample, global mean normalization was performed calculating the ΔCt values for each miRNA (ΔCt = Ct experiment miRNA – mean Ct endogen miRNA in all assessed miRNAs). Then, ΔΔCt was calculated (ΔCt mean target miRNA - ΔCt mean controls miRNAs). Benjamin-Hochberg correction test was applied as an estimated false discovery rate (FDR of 5%). Samples were compared by their miRNA relative expression profiles between PCs and TCs ($2^{-\Delta Ct}$). Fold change (log_2) expression of differentially expressed miRNAs in PCs and TCs were calculated as $2^{-\Delta\Delta Ct}$. A fold change scale of 1-infinity would indicate an upregulation, whereas a scale of 0-1 would represent a downregulation. Heatmap of hierarchical clustering comparing TCs and PCs, by their miRNA expression profiles was performed. The Euclidean distance-metric hierarchical cluster represented the relative expression of upregulated miRNAs in green tones

and downexpressed miRNAs in red tones. The diagnostic accuracy for predicting individuals belonging to TCs or PCs group was evaluated by logistic regression and receiver operating characteristic (ROC) curve analysis.

The statistical software used was the SPSS Software v22 and XLSTAT 2020.5.1.1064. Representations of graphs were performed using GraphPad Prism 5.0 software, XLSTAT and BioRender Online Software. miRNAs interaction network representations were generated using the miRNet visual analytics database, version 2.0 web server [16]. The results were considered statistically significant at $P<0.05$.

RESULTS AND DISCUSSION

To our knowledge, this is the first study to evaluate circulating miRNA predictive markers and disease progression between these phenotypical groups of ECs. Our results showed a consistent difference in plasma miRNA expression between the PCs and TCs before the loss of viral control. These differences in the miRNA expression patterns persisted after the loss of control, showing that there is an association between the miRNA profile and viraemia. Interestingly, we discovered that most of these differentially expressed miRNAs are involved in lipid metabolism. This finding confirms our previous results that revealed a strong correlation between circulating metabolites and lipid concentrations with immunological parameters[17], offering new insight into metabolic pathways associated with spontaneous loss of viral control.

Characteristics of the study participants

The clinical and demographic characteristics of persistent controllers (PCs) and transient controllers (TCs) before the loss of viral control under the pre-loss condition are summarized in Table S1. No differences were observed in age, sex, transmission route, HCV coinfection, or $CD4^+$ and $CD8^+$ T-cell counts in the TCs compared to the PCs.

Table S1. Baseline characteristics of the study participants.

Clinical characteristics	PCs (n=6)	TCs (n=12)	P-value
Age (years)	47 [46-51]	44 [38-57]	0.335
Male	2 (33.3)	6 (50.0)	0.616
Risk factor			0.277
Heterosexual	2 (33.3)	5 (41.7)	
Homo/Bisexual		2 (16.7)	
Intravenous drug abuse	4 (66.7)	3 (25.0)	
Other/Unknown		2 (16.7)	
$CD4^+$ T-cell count (cells/µL)	424 [382-674]	712 [418-913]	0.213
$CD8^+$ T-cell count (cells/µL)	350 [256-770]	764 [636-1200]	0.090
$CD4^+$/$CD8^+$ T cell ratio	1.45 [0.74-1.99]	0.93 [0.66-1.07]	0.320
HCV co-infection (Positive)	3 (50.0)	5 (41.7)	0.563

Data from TCs corresponds to one measurement in the period of two years before the loss of virological control (–T24 to –T0), compared to PCs. Data are presented as n (%) or median [interquartile range]. Categorical data were compared through a $\chi 2$ test, whereas continuous data were compared using the non-parametric Mann-Whitney test. PCs, persistent controllers; TCs, transient controllers.

Circulating miR-27a-3p, miR-376a-3p and miRNA-199a-3p as predictive markers of the loss of viral control in ECs

TCs showed an upregulated miRNA profile before the loss of viral control, compared to PCs. Differential miRNA expression between TC and PC groups was measured using the $2^{-\Delta\Delta Ct}$ method (fold-change (FC) relative to that of the PCs). From the 188 miRNAs analysed in this study, we found 23 miRNAs differentially expressed in TCs compared to PCs at the pre-loss time point (P<0.05) (Figure 1, Table S2). The most highly expressed miRNAs (FC>4.0) were hsa-miR-27a-3p, hsa-miR-376a-3p and hsa-miR-199a-3p, which exhibited 4.8-, 4.2- and 4.0-fold increased expression, respectively, in the TCs (Figure1B).

Table S2. Significantly expressed miRNAs in TCs compared to PCs under the pre-loss condition.

miRNA	Relative Expression PCs	Relative Expression TCs	P-value	Fold Change
**hsa-miR-27a-3p**	**0.54**	**2.57**	**0.002**	**4.8**
**hsa-miR-376a-3p**	**0.01**	**0.05**	**0.009**	**4.2**
**hsa-miR-199a-3p**	**0.08**	**0.34**	**0.013**	**4.0**
hsa-miR-423-3p	0.03	0.09	0.018	3.3
hsa-miR-146a-5p	0.12	0.38	0.026	3.1
hsa-miR-584-5p	0.11	0.34	0.016	3.1
hsa-miR-148b-3p	0.03	0.08	0.020	3.0
hsa-miR-191-5p	0.02	0.07	0.020	2.8
hsa-miR-335-5p	0.18	0.51	0.017	2.8
hsa-miR-22-3p	0.62	1.69	0.002	2.7
hsa-miR-142-5p	0.13	0.34	0.036	2.6
hsa-miR-324-5p	0.01	0.02	0.045	2.6
hsa-let-7d-3p	0.09	0.22	0.038	2.5
hsa-miR-424-5p	0.05	0.13	0.005	2.5
hsa-miR-484	0.12	0.31	0.003	2.5
hsa-miR-33a-5p	0.02	0.04	0.040	2.3
hsa-miR-148a-3p	0.05	0.11	*0*.020	2.2
hsa-miR-26b-5p	0.25	0.52	0.006	2.1
hsa-miR-423-5p	0.21	0.43	0.013	2.0
hsa-miR-140-3p	0.02	0.04	0.037	1.9
hsa-miR-29b-3p	0.04	0.08	0.020	1.9
hsa-miR-26a-5p	0.10	0.17	0.042	1.7
hsa-miR-93-5p	0.06	0.10	0.037	1.7

Data values of the significantly expressed miRNAs (relative expression) of PCs and TCs under the pre-loss condition are represented by the fold change number (highest to lowest FC). In bold are represented the significantly upregulated miRNAs (FC>4).

To test whether these three miRNAs can distinguish TCs from PCs, logistic regression and receiver operating characteristic (ROC) curve analysis were carried out. Thus, we used a logistic regression model that combined 3 significant miRNAs (hsa-miR-27a-3p, hsa-miR-376a-3p and hsa-miR-199a-3p), which displayed an area under the curve (AUC) with a value of 1 (SE=100, P< 0.05) (Figure 1C).

Finally, we evaluated which metabolic pathways were affected by the interaction of these three significantly expressed miRNAs in TCs. Thus, some of the most relevant functions of hsa-miR-27a-3p involve lipid metabolism (P=0.129), adipocyte differentiation (P=0.114), cholesterol metabolism (hydrolysis, influx and esterification) (P<0.001) and cholesterol efflux

(P=0.043), whereas hsa-miR-376a-3p is associated with glycosphingolipid biosynthesis (*B3GALT5,* P=0.072). The liver miRNA hsa-miR-199a-3p is interestingly associated with HIV lipodystrophy (P=0.047).

Considering that an upregulated miRNA plasma profile is associated with spontaneous loss of viral control in ECs, we analysed the relationship between these specific pre-loss miRNA patterns and clinical parameters (Table S1). The analysis was performed considering the entire patient cohort (n=18) using the relative expression value of each differentially expressed miRNA as calculated by the $2^{-\Delta Ct}$ method. All 23 miRNAs differentially expressed in TCs under the pre-loss condition were positively correlated with VL except for miR-26b-5p (Table S3). Additionally, 7 miRNAs (miR-146a-5p, miR-199a-3p, miR-22-3p, miR-26a-5p, miR-324-5p, miR-423-3p and miR-93-5p) were positively correlated with $CD4^+$ T-cell counts, and 11 miRNAs (miR-142-5p, miR-146a-5p, miR-148a-3p, miR-148b-3p, miR-199a-3p, miR-26b-5p, miR-29b-3p, miR-335-5p, miR-424-5p, miR-484 and miR-584-5p) were positively correlated with $CD8^+$ T-cell counts (Table S3). Additionally, miRNA hsa-miR-424-5p was negatively associated with the CD4/CD8 ratio (P=0.007, ρ=-0.704). No association was found between the selected miRNAs and sex or risk factors. Interestingly, hsa-miR-199a-3p, which was expressed at a 4.0-fold higher level in TCs than in PCs before the loss of viral control, was positively correlated with VL, $CD4^+$ and $CD8^+$ T-cell counts under the pre-loss condition (P=0.002, ρ=0.779, P=0.015 ρ=0.654 and P=0.042 ρ=0.620, respectively).

Table S3. Pre-loss miRNA profile correlates with plasma viraemia rebound, $CD4^+$ and $CD8^+$-T cells.

	log VL (copies/ml)		$CD4^+$ counts		$CD8^+$ counts	
**miRNA**	**P-value**	**ρ**	**P-value**	**ρ**	**P-value**	**ρ**
hsa-let-7d-3p	**0.000**	**0.784**	0.513	0.176	0.081	0.482
hsa-miR-140-3p	**0.016**	**0.590**	0.704	0.103	0.111	0.444
hsa-miR-142-5p	**0.000**	**0.880**	0.122	0.433	**0.046**	**0.561**
hsa-miR-146a-5p	**0.008**	**0.654**	**0.009**	**0.649**	**0.006**	**0.718**
hsa-miR-148a-3p	**0.008**	**0.637**	0.542	0.165	**0.010**	**0.662**
hsa-miR-148b-3p	**0.001**	**0.729**	0.196	0.341	**0.046**	**0.541**
hsa-miR-191-5p	**0.005**	**0.661**	0.090	0.437	0.337	0.278
hsa-miR-199a-3p	**0.002**	**0.779**	**0.015**	**0.654**	**0.042**	**0.620**
hsa-miR-22-3p	**0.000**	**0.879**	**0.031**	**0.599**	0.105	0.515
hsa-miR-26a-5p	**0.001**	**0.745**	**0.046**	**0.521**	0.156	0.400
hsa-miR-26b-5p	**0.066**	**0.525**	0.144	0.429	**0.043**	**0.592**
hsa-miR-27a-3p	**0.001**	**0.811**	0.217	0.385	0.109	0.510
hsa-miR-29b-3p	**0.000**	**0.794**	0.289	0.282	0.056	0.521
hsa-miR-324-5p	**0.035**	**0.611**	**0.022**	**0.650**	0.121	0.473
hsa-miR-335-5p	**0.010**	**0.767**	0.511	0.236	**0.035**	**0.703**
hsa-miR-33a-5p	**0.018**	**0.621**	0.366	0.262	0.477	0.228
hsa-miR-376a-3p	**0.014**	**0.619**	0.206	0.346	0.373	0.270
hsa-miR-423-3p	**0.001**	**0.772**	**0.010**	**0.662**	0.068	0.502
hsa-miR-423-5p	**0.000**	**0.817**	0.098	0.459	0.087	0.492
hsa-miR-424-5p	**0.029**	**0.562**	0.732	-0.096	**0.006**	**0.718**
hsa-miR-484	**0.000**	**0.863**	0.213	0.329	**0.016**	**0.627**
hsa-miR-584-5p	**0.031**	**0.558**	0.248	0.318	**0.009**	**0.669**
hsa-miR-93-5p	**0.001**	**0.751**	**0.034**	**0.532**	0.277	0.312

P and ρ values of the Spearman correlation between significantly expressed miRNAs, VL (copies/mL), $CD4^+$ and $CD8^+$-T cell counts in TCs under the pre-loss condition. In bold are represented the significant correlations.

Hsa-miR-27a-3p, hsa-miR-376a-3p and hsa-miR-199a-3p were the most highly expressed miRNAs in TCs compared to those expressed in the PCs under the pre-loss condition. Furthermore, the spontaneous loss of viral control in ECs can be defined at several levels concerning these miRNAs, as shown by the diagnostic accuracy determined by the ROC analysis. A previous study has reported differences between HIV-infected patients showing disease progression (viraemic progressors, VP) and ECs, anticipating that specific miRNA profile could influence the different progression of HIV disease. Of interest, the study identified the upregulation of hsa-miR-27a, -27b and 29b levels in ECs (FC>2.1) and concluded that the miRNA profile of ECs is similar to healthy individuals and differs from VP [7]. As we previously stated, some miRNAs play important roles in the regulation of biological processes, for example, the overexpression of hsa-miR-27a can decrease the phosphorylation of Akt and ERK, and this antiviral activity can inhibit enterovirus 71 (EV71) replication[18]. Other studies have reported the proinflammatory activity of hsa-miR-27a; when this miRNA is overexpressed, the expression of proinflammatory cytokines is increased via TLR2/4-activated macrophages[19]. Of note, during the immune activation for viral replication, the high energy demand is characterized by the overproduction of proinflammatory cytokines as suggested in our previous works[4,20], leading to an inflammatory and increased oxidative stress condition in PLWH [21]. The activation of a cytokine deregulation in TCs may result in metabolic alterations that could be associated with the dysregulation of miRNA expression, such as through the combination of hsa-mir-376a-3p and hsa-miR-16-5p (miR-376a-3p/miR-16-5p) that can be detected to distinguish cognitive impairment (CI) groups from non-CI groups of PLWH[22], or the liver-specific miRNA hsa-miR-199a-3p which is upregulated to a significant degree (FC=2.5, P<0.01) in HIV-infected subjects with lipodystrophy[23], a medical condition characterized by the loss of fat tissue from one body area. Moreover, subcutaneous adipose tissue of HIV-infected patients has an overproduction of inflammatory cytokines[24] and lipogenesis, the metabolic formation of fat, could be the result of immune dysregulation promoted by ART[25].

Plasma viraemia influences the miRNA profile of ECs

We also evaluated the plasma miRNA profile in TCs under the post-loss condition (from T0 to T+12) and it to that of the control group of PCs (Table S4). Overall, TCs under the post-loss condition also showed an upregulated miRNA profile compared to that of the PCs, except for hsa-miR-497-5p, which showed downregulated expression in the TCs compared to the PCs. In this case, the most highly expressed miRNAs (FC>5.0) in the TCs compared to the PCs were hsa-miR-27b-3p, hsa-miR-326, hsa-miR-339-5p, hsa-miR-324-5p, hsa-miR-543, hsa-miR-495-3p and hsa-miR-199a-3p (Figure 2A).

Because hsa-miR-199a-3p and hsa-miR-27a-3p had been previously connected to lipid metabolism (Figure 1D), we considered probed the association of these significantly expressed miRNAs under the post-loss condition with relevant genes linked to lipid pathways (Figure 2B).

Then, to determine whether any of these differentially expressed miRNAs can be used for clinically monitoring ECs, we determined the correlation between miRNA relative expression levels ($2^{-\Delta Ct}$) and the VL and the $CD4^+$ and $CD8^+$ T-cell counts. The relative expression levels of hsa-miR-130a-3p, hsa-miR-130b-3p, hsa-miR-140-3p, hsa-miR-199a-3p, hsa-miR-22-3p, hsa-miR-27a-3p, hsa-miR-27b-3p, hsa-miR-320b, hsa-miR-423-3p and hsa-miR-584-5p were positively associated with VL. Additionally, the expression levels of hsa-miR-24-3p, hsa-miR-30e-3p and hsa-miR-339-5p increased with increasing $CD4^+$ T-cell counts, whereas the

expression of hsa-miR-126-3p, hsa-miR-146a-5p, hsa-miR-335-5p, hsa-miR-339-5p and hsa-miR-423-3p increased with increasing CD8$^+$ T-cell counts (Table S5).

Interestingly, the relative expression of hsa-miR-497-5p, which was the only miRNA significantly downregulated in the TCs compared to the PCs, was negatively correlated with both VL and CD8$^+$ T-cell counts (P=0.023, ρ=-0.646 and P=0.010 ρ=-0.706, respectively).

Table S4. Significantly expressed miRNAs in TCs compared to PCs under the post-loss condition.

miRNA	Relative Expression PCs	Relative Expression TCs	P-value	Fold Change
**hsa-miR-27b-3p**	**0.17**	**1.24**	**0.004**	**7.2**
**hsa-miR-326**	**0.03**	**0.18**	**0.005**	**6.7**
**hsa-miR-339-5p**	**0.01**	**0.04**	**0.036**	**5.7**
**hsa-miR-324-5p**	**0.01**	**0.03**	**0.012**	**5.5**
**hsa-miR-543**	**0.00**	**0.02**	**0.024**	**5.4**
**hsa-miR-495-3p**	**0.00**	**0.02**	**0.048**	**5.1**
**hsa-miR-199a-3p**	**0.08**	**0.43**	**0.003**	**5.0**
hsa-miR-221-3p	0.27	1.31	0.001	4.9
hsa-miR-191-5p	0.02	0.11	0.027	4.7
hsa-miR-423-3p	0.03	0.12	0.003	4.6
hsa-miR-146a-5p	0.12	0.56	0.004	4.5
hsa-miR-126-3p	0.40	1.70	0.001	4.2
hsa-miR-130a-3p	0.08	0.33	0.010	4.1
hsa-miR-148b-3p	0.03	0.10	0.015	4.1
hsa-miR-335-5p	0.18	0.76	0.003	4.1
hsa-miR-130b-3p	0.02	0.09	0.022	3.8
hsa-miR-376a-3p	0.01	0.04	*0.055*	3.5
hsa-miR-22-3p	0.62	2.07	0.002	3.3
hsa-miR-151a-5p	0.06	0.19	0.048	3.2
hsa-miR-22-5p	0.01	0.05	0.013	3.2
hsa-miR-24-3p	0.08	0.27	0.015	3.2
hsa-miR-27a-3p	0.54	1.73	0.001	3.2
hsa-let-7d-3p	0.09	0.29	0.036	3.1
hsa-miR-142-5p	0.13	0.41	0.035	3.1
hsa-miR-148a-3p	0.05	0.16	0.037	3.1
hsa-miR-128-3p	0.02	0.04	0.042	2.9
hsa-miR-584-5p	0.11	0.31	0.005	2.8
hsa-miR-425-5p	0.01	0.03	0.015	2.7
hsa-miR-29b-3p	0.04	0.10	0.011	2.5
hsa-miR-30d-5p	0.08	0.19	0.020	2.5
hsa-miR-30e-3p	0.01	0.03	0.024	2.5
hsa-miR-484	0.12	0.30	0.016	2.4
hsa-miR-93-5p	0.06	0.15	0.015	2.4
hsa-miR-140-3p	0.02	0.04	0.042	2.0
hsa-miR-320b	0.18	0.33	0.036	1.9
hsa-miR-423-5p	0.21	0.39	0.040	1.8
hsa-miR-19a-3p	0.25	0.43	0.048	1.7
**hsa-miR-497-5p**	**0.09**	**0.03**	**0.008**	**0.3**

Data values of the significantly expressed miRNA (relative expression) of PCs and TCs under the post-loss condition are represented by the fold change number (highest to lowest FC). In bold are represented the significantly upregulated miRNAs (FC>5) and the downregulated miRNAs at the bottom of the table (FC<0.4).

Table S5. Post-loss miRNA profile correlates with plasma viraemia rebound, $CD4^+$ and $CD8^+$-T cells.

	log VL (copies/ml)		CD4+ counts		CD8+ counts	
**miRNA**	**P-value**	**ρ**	**P-value**	**ρ**	**P-value**	**ρ**
hsa-let-7d-3p	0.105	0.435	0.603	0.146	0.308	0.282
hsa-miR-126-3p	0.089	0.490	0.803	0.077	**0.026**	**0.636**
hsa-miR-128-3p	0.111	0.414	0.259	0.300	0.248	0.318
hsa-miR-130a-3p	**0.039**	**0.505**	0.220	0.314	0.269	0.294
hsa-miR-130b-3p	**0.023**	**0.564**	0.444	0.206	0.140	0.400
hsa-miR-140-3p	**0.044**	**0.508**	0.222	0.324	0.327	0.262
hsa-miR-142-5p	0.338	0.289	0.297	0.313	0.058	0.538
hsa-miR-146a-5p	0.129	0.464	0.131	0.462	**0.001**	**0.839**
hsa-miR-148a-3p	0.131	0.382	0.729	-0.091	0.158	0.371
hsa-miR-148b-3p	0.241	0.301	0.141	0.373	0.188	0.347
hsa-miR-151a-5p	0.483	0.183	0.379	0.228	0.134	0.391
hsa-miR-191-5p	0.402	0.218	0.722	0.093	0.295	0.279
hsa-miR-199a-3p	**0.016**	**0.630**	0.140	0.415	0.091	0.468
hsa-miR-19a-3p	0.176	0.418	0.101	0.497	0.142	0.473
hsa-miR-221-3p	0.092	0.486	0.188	0.390	0.162	0.412
hsa-miR-22-3p	**0.007**	**0.703**	0.201	0.379	0.209	0.374
hsa-miR-22-5p	0.083	0.462	0.071	0.479	0.147	0.393
hsa-miR-24-3p	0.086	0.429	**0.035**	**0.515**	0.305	0.274
hsa-miR-27a-3p	**0.028**	**0.565**	0.676	0.118	0.246	0.332
hsa-miR-27b-3p	**0.015**	**0.681**	0.297	0.329	0.075	0.531
hsa-miR-29b-3p	0.178	0.354	0.356	0.247	0.393	0.229
hsa-miR-30d-5p	0.174	0.346	0.191	0.333	0.165	0.365
hsa-miR-30e-3p	0.631	0.186	**0.050**	**0.667**	0.125	0.550
hsa-miR-320b	**0.021**	**0.590**	0.899	0.036	0.232	0.329
hsa-miR-324-5p	0.224	0.347	0.114	0.442	0.185	0.376
hsa-miR-326	0.071	0.463	0.520	0.174	0.269	0.294
hsa-miR-335-5p	0.077	0.529	0.191	0.406	**0.039**	**0.601**
hsa-miR-339-5p	0.112	0.428	**0.020**	**0.593**	**0.044**	**0.525**
hsa-miR-376a-3p	0.742	0.093	0.451	0.211	0.970	0.011
hsa-miR-423-3p	**0.026**	**0.555**	0.418	0.218	**0.011**	**0.632**
hsa-miR-423-5p	0.222	0.335	0.467	0.204	0.694	0.111
hsa-miR-425-5p	0.221	0.313	0.940	0.020	0.176	0.356
hsa-miR-484	0.123	0.402	0.305	0.274	0.444	0.206
hsa-miR-495-3p	0.460	0.236	0.443	0.245	0.433	0.264
hsa-miR-497-5p	**0.023**	**-0.646**	0.417	-0.259	**0.010**	**-0.706**
hsa-miR-543	0.420	0.235	0.513	-0.191	0.803	0.077
hsa-miR-584-5p	**0.032**	**0.537**	**0.070**	**0.465**	0.165	0.365
hsa-miR-93-5p	0.241	0.301	0.633	0.125	0.499	0.182

P and ρ values of the Spearman correlation between significantly expressed miRNAs, VL (copies/mL), $CD4^+$ and $CD8^+$-T cell counts in TCs under post-loss condition. In bold are represented the significant correlations.

Circulating miRNA 199a-3p predicts plasma viraemia rebound

Next, we searched for circulating miRNAs capable of predicting the loss of viral control (the pre-loss condition) that remained differentially expressed during viraemia rebound (the post-loss condition). A Venn diagram (Figure 3A) displays the number and overlap of significantly differentially expressed miRNAs in TCs compared to PCs under both conditions. Thus, these 19

mRNAs were significantly upregulated in the TCs over time under both conditions compared to the PCs and can be considered potential biomarkers of the loss of natural control in EC. Notably, the expression of hsa-miR-199a-3p, hsa-miR-376a-3p and hsa-miR-27a-3p, which were the most highly expressed miRNAs in the TCs compared to the PCs (FC=4.0) before the spontaneous loss of viral control (Figure 1B), remained significantly upregulated after the loss of viral control. Indeed, the expression of hsa-miR-199a-3p was highly upregulated in the TCs under both conditions before (FC>4) and after (FC>5) the loss of viral control. Thus, we carried out an analysis by using logistic regression and receiver operating characteristic ROC curves of only hsa-miR-199a-3p under both conditions to evaluate its discriminatory power *per se*. The model displayed an area under the curve (AUC) value of 0.896 (SE=75%, P=0.014) before loss of viral control and an AUC value of 0.958 (SE=100%, P=0.05) after loss of control. These results suggest that this miRNA showed an optimal percentage of separation and can be used to distinguish EC, PC and TC groups under both conditions (Figure 3B).
Interestingly, some of the target sites of the circulating differentially expressed miRNAs under the pre-and post-condition are located in viral RNA regions encoding viral accessory proteins, modulating HIV replication. Interestingly, two of the significantly expressed miRNAs in TCs under the post-loss condition, hsa-miR-29b and hsa-miR-326, target the *nef* gene, which is important in delayed disease progression. Besides, the inhibitory effect of HIV replication by the hsa-miR-29a/29b miRNA can be a mechanism against the virus after losing viral control in TCs. Another remarkable miRNA target in the HIV genome is the *gag* gene, targeted by hsa-miR-423-3p, the *vpr* gene targeted by hsa-miR-149 and the *vif* gene targeted by hsa-miR-324-5p [26] (Figure 4).

HIV-1 is one of the many viruses whose expression has been demonstrated to be modulated by cellular miRNAs[27]. miRNAs can affect HIV replication by directly targeting HIV RNA or targeting some of the cell factors necessary for its replication, suggesting that HIV infection strongly influences the plasma miRNA profile. Interestingly, our results confirmed differences in the expression of some of these miRNAs, before and after the loss of viral control, with target sites located in viral RNA regions encoding viral accessory proteins. The miRNAs that can directly decrease HIV replication by more than 40%[28], hsa-miR-423-3p, hsa-miR-29b-3p and hsa-miR-324-5p, were significantly upregulated in the TCs compared to the PCs under both conditions, whereas hsa-miR-326 was one of the most upregulated miRNAs in the TCs after the spontaneous loss of viral control. Notably, as mentioned above, hsa-miR-423-3p targets the *gag* gene, hsa-miR-29a/29b and hsa-miR-326 target the *nef* gene, and hsa-miR-324-5p targets the *vif* gene[26,29] (Figure 4). The expression of hsa-miR-423-3p was previously reported to be an oncogene in several types of cancers. In fact, overexpression of hsa-miR-423-3p was postulated to be an indicator of poor prognosis in patients with lung cancer, where it seems to promote cell proliferation, migration and invasion[30]. There is no evidence of a relationship between hsa-miR-423-3p and viral load, but according to these previous findings, our current results suggest that overexpression of hsa-miR-423-3p may be an indicator of poor disease prognosis in TCs, possibly associated with a proinflammatory profile. Recently, the expression of hsa-miR-324-5p was associated with host defence against the highly pathogenic influenza A virus (H5N1 HPAIV), suppressing PB1 expression and targeting CUEDC2, a negative regulator of the JAK-STAT pathway with subsequent viral replication[31]. Both hsa-miR-29b and hsa-miR-326 have been previously reported as regulators of Th1 and Th17 differentiation, respectively.

Indeed, the expression of hsa-miR-29b was previously identified as a regulator of Th1 differentiation and acts by controlling T-box 21 protein (T-bet) transcription factors and IFN-γ. Hsa-miR-29b is increased in memory $CD4^+$ T-cells obtained from multiple sclerosis (MS) patients and IFN-γ itself enhances hsa-miR-29b expression and establishes a regulatory feedback loop[32]. Besides, hsa-miR-29b, in combination with hsa-miR-27b, can inhibit the expression of Cyclin T1 in resting $CD4^+$ T cells[33]. On the other hand, hsa-miR-29 binds directly to the HIV-1 mRNA increasing its association with proteins in the RISC complex which leads to the inhibition of the translation of viral proteins and viral replication[34]. Hsa-miR-326 is highly expressed in Th17 cells compared with other subtypes of $CD4^+$ T cells in relapsing-remitting multiple sclerosis patients and promotes Th-17 differentiation inhibiting ETS-1, a negative regulator of Th-17 differentiation[35].

Interestingly, before the loss of viral control, the expression of no miRNAs was downregulated in the TCs compared to the PCs, indicating that the profiles of miRNAs of these patients indicate high activity. In contrast, after the spontaneous loss of viral control, our results revealed the downregulation of the expression of miR-497-5p. This miRNA belongs to the microRNA-15/16/195/424/497 family cluster and plays an important role in modulating the cell cycle and apoptosis of infected cells[33,36]. It targets the BCL2L2 gene, which encodes an anti-apoptotic protein (Bcl-2-like protein 2), and the SMAD2 gene (encoding the SMAD family member 2 protein), both of which play important roles in modulating intrinsic apoptotic pathways, the cell cycle and multiple cellular processes [37]. Additionally, the expression of hsa-miR-497-5p was suggested to inhibit the proliferation, migration and invasion of colon cancer cells (CRC) by negatively regulating the insulin-like growth factor 1 [38]. It has been reported that miR-497-5p targets Pur-α, a cellular partner for the Tat regulatory protein of HIV-1, which is necessary for HIV-1 infection of macrophages [39]. Although this mechanism is still unknown, the modulation of hsa-miR-497-5p activity may decrease HIV transcription in macrophages [33,40,41]. Accordingly, the downregulation of hsa-miR-497-5p may enhance the HIV transcription and consequently the spontaneous loss of viral control and disease progression in TCs.

Viraemia effects on the TCs miRNA profile

The VL evolution from TCs under the post-loss condition was 558 [133-983] at +T0 and 1750 [142-8185] HIV-RNA copies/mL under the post-loss condition at 12 month time-point (median [interquartile range]).

After discovering differences in the miRNA plasma profiles between TCs and PCs over time, to further identify the specific influence of viral rebound in the plasma miRNA profile, we compared the relative miRNA expression between the pre- and post-loss conditions for each TC by applying the Wilcoxon test for paired samples. A total of 16 miRNAs were differentially expressed between these conditions (Table S6). The expression levels of hsa-miR-144-3p, hsa-miR-497-5p and hsa-miR-122-5p were significantly downregulated, and the expression of hsa-miR-27b-3p, hsa-miR-130b-3p and hsa-miR-326 was significantly upregulated in the TCs when both conditions were compared. Notably, differences in hsa-miR-27b-3p, hsa-miR-326 and hsa-miR-497-5p expression were previously found to be significantly different between TCs and PCs under the post-loss condition. These results confirmed that these three specific miRNAs are remarkably associated with viraemia rebound in TCs and therefore may be relevant predictors of the spontaneous loss of viral control in a cohort of ECs, discriminating between PCs and TCs.

Table S6. miRNA profile in TCs before and after the loss of viral control.

miRNA	Relative Expression (pre-loss)	Relative Expression (post-loss)	P-value	Fold Change
**hsa-miR-326**	**0.05**	**0.183**	***0.051***	**3.4**
**hsa-miR-130b-3p**	**0.03**	**0.090**	**0.028**	**3.3**
**hsa-miR-27b-3p**	**0.45**	**1.235**	**0.046**	**2.8**
hsa-miR-324-5p	0.01	0.033	0.018	2.6
hsa-miR-339-5p	0.02	0.038	0.012	2.5
hsa-miR-425-5p	0.01	0.029	0.005	2.5
hsa-miR-130a-3p	0.16	0.331	0.047	2.0
hsa-miR-24-3p	0.14	0.272	0.047	2.0
hsa-miR-151a-3p	0.04	0.070	0.038	1.8
hsa-miR-421	0.01	0.026	0.046	1.8
hsa-miR-93-5p	0.08	0.147	0.037	1.8
hsa-miR-191-5p	0.07	0.112	0.037	1.5
hsa-miR-301a-3p	0.02	0.026	0.043	1.3
**hsa-miR-122-5p**	**0.07**	**0.041**	**0.028**	**0.6**
**hsa-miR-144-3p**	**0.52**	**0.231**	**0.018**	**0.4**
**hsa-miR-497-5p**	**0.07**	**0.030**	**0.046**	**0.4**

Data values of the significantly expressed miRNAs (relative expression) of TCs under the pre- and post-loss condition are represented by the fold change number (highest to lowest FC). In bold are represented the significantly upregulated miRNAs (FC>2.5) and the downregulated miRNAs in TCs, at the top and the bottom of the table, respectively (FC<0.5).

Viraemia induces an upregulated LDL metabolism in TCs

Because we found that the miRNAs with the highest expression in TCs compared to PCs under both conditions (pre- and post-loss conditions) (miR-199a-3p, miR-27a-3p, miR-27b-3p, miR-324-5p, miR-326, miR-339-5p, miR-376a-3p, miR-495-3p and miR-543) were strongly related to lipid and lipoprotein metabolism, we further investigated the potential roles of lipoproteins in the ECs. First, based on 1H-NMR spectroscopy, no statistically significant differences in lipoprotein levels were found between the TCs and PCs, neither before nor after the loss of viral control (non-parametrical Mann-Whitney U test). On the other hand, alterations in LDL metabolism, including significant changes in LDL composition (LDL cholesterol, P=0.013) LDL size particles (LDL-P, P=0.008), and among LDL-large, medium and small particles (P=0.021, P=0.004 and P=0.026, respectively), were found to be significantly increased in the TCs after the loss of viral control compared to their baseline values (pre-loss condition) by applying the Wilcoxon test for paired samples. In addition, both non-HDL particles (P=0.010) and the LDL-P/HDL-P ratio (P=0.075), which is an important predictor of cardiovascular risk, were increased with viraemia. Thus, our results suggested that LDL metabolism is closely associated with metabolic disorders that can be related to the loss of viral control (Figure S2).

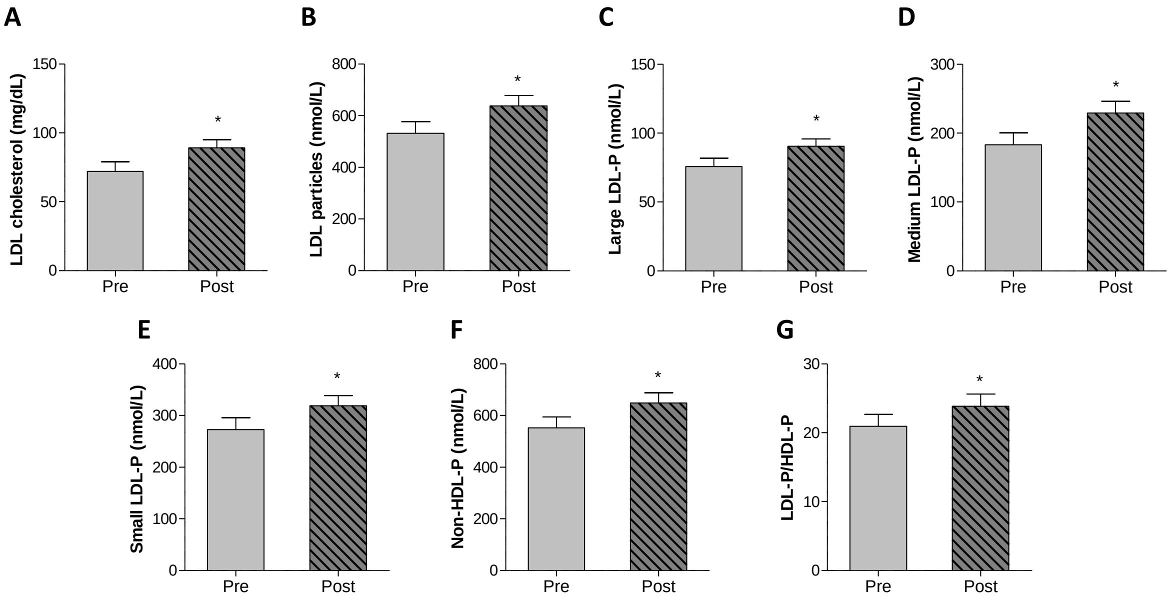

Figure S2. Representation of the significant changes in LDL composition in TCs after the loss of viral control compared to their baseline values (pre-loss condition) applying the Wilcoxon test for paired samples. Significant p-values in LDL cholesterol metabolism: **A)** LDL-Cholesterol (P=0.013), **B)** LDL size particles (P=0.008), among **C)** LDL-large, **D)** medium and **E)** small particles (P=0.021, P=0.004 and P=0.026, respectively) were found significantly increased in TCs after the loss of viral control. **F)** Non-HDL particles and **G)** LDL-P/HDL-P ratio resulted to be increased with viraemia (P=0.010 and P=0.075, respectively).

Therefore, we evaluated the association between the highly expressed miRNAs in the TCs compared to the PCs under the two conditions and the associated lipid and lipoprotein profiles. Interestingly, the liver-specific miRNAs hsa-miR-199a-3p and hsa-miR-376a-3p were positively associated with LDL and HDL metabolism, respectively, in the TCs under the pre-loss condition. Hsa-miR-199a-3p was significantly associated with LDL particles (P=0.047, ρ=0.714), specifically small and medium LDL particles (P=0.047 and P=0.028, respectively), whereas the miRNA hsa-miR-376a-3p was significantly correlated with HDL triglycerides (HDL-TG) (P=0.016, ρ=0.700), HDL particles (P=0.029, ρ=0.655), and specifically small HDL particles (P=0.023, ρ=0.673) in the TCs under the pre-loss condition. However, hsa-miR-27a-3p, which was highly upregulated in the TCs before the loss of viral control (FC=4.8), was negatively correlated with LDL particles (P=0.042, ρ=-0.829) under the pre-loss condition. In contrast, hsa-miR-497-5p, the only miRNA downregulated in the TCs after the loss of viral control, was positively associated with LDL metabolism, LDL particles (P=0.021, ρ=0.786) and LDL small particles (P=0.037, ρ=0.738) and with HDL metabolism, specifically with HDL particles (P=0.037, ρ=0.738), in the TCs under the post-loss condition (Figure S3).

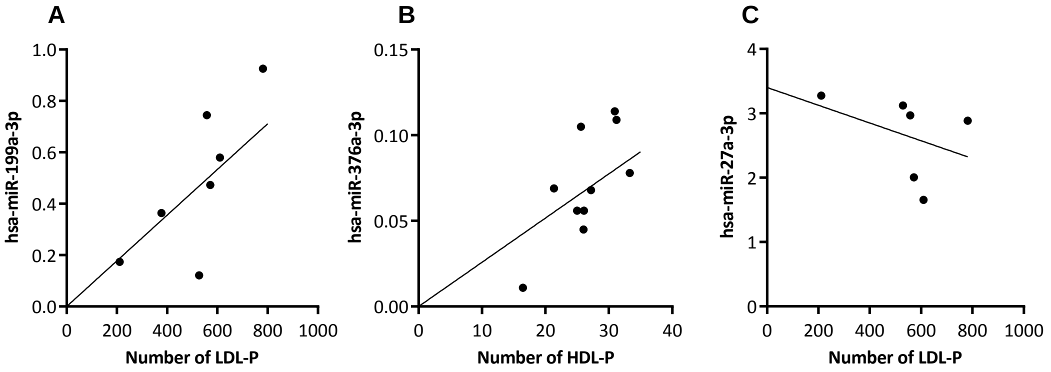

Figure S3. Significantly upregulated miRNAs in TCs under the pre-loss condition correlates with LDL and HDL metabolism. Spearman correlation of the significantly upregulated miRNAs in TCs under the pre-loss condition **A)** hsa-miR-199a-3p (P=0.047, ρ=0.714), **B)** hsa-miR-376a-3p (P=0.029, ρ=0.655) and **C)** hsa-miR-27a-3p (P=0.042, ρ=-0.829) and LDL and HDL particles.

Several miRNAs can regulate different steps of HDL-C metabolism, from synthesis to clearance [42,43], including cellular cholesterol efflux, HDL biogenesis, and HDL uptake [44]. Additionally, recent studies have promoted the importance of miRNAs in controlling plasma LDL-C levels, cholesterol biosynthesis, hepatic LDL receptor (LDLR) expression and in the regulation of genes involved in very-low-density lipoprotein (VLDL) secretion[45]. Surprisingly, hsa-miR-27, one of the most upregulated miRNA in the current work, is one of the key regulators of the expression of ABCA1 and the LDL receptor [46] and was previously found to be involved in the regulation of adipogenesis [47] and cholesterol biosynthesis [48] (Figure 1C). Hsa-miR-122, identified in plasma exosomes of HIV/HCV patients, can bind directly to the 5'-UTR of hepatitis virus (HCV) RNA by promoting its replication[49] and similar to hsa-miR-199a-3p, is implicated in fatty acid and cholesterol biosynthesis[50]. Overexpression of miR-130b-3p or miR-130b-5p in HepG2 cells increases the secretion of very-low-density lipoprotein (VLDL) particles, enhances the secretion of glycerol metabolically labelled triglyceride (TG), and significantly increases the number or the average size of lipid droplets (LDs), respectively[51]. Thus, our results suggested that disturbance in lipoprotein levels, mostly induced by upregulation of some liver-specific miRNAs (such as hsa-miR-199a-3p), may be highly associated with the immunological factors behind the loss of viral control in TCs.

In conclusion, we demonstrate that a specific miRNA pattern in ECs may be used as a biomarker for quick screening of the virological and immunological progression in ECs, and we confirmed that viraemia induces increased LDL metabolism in TCs under both pre-loss and post-loss conditions. The association between transcriptomics and previously used omics studies was also challenging because of the multiple different genes that can be affected by a single miRNA and all the biological pathways involved.

REFERENCES

1. Dominguez-Molina B, Leon A, Rodriguez C, et al. Analysis of non-AIDS-defining events in HIV controllers. *Clin Infect Dis*. 2016;62(10):1304-1309. doi:10.1093/cid/ciw120
2. Leon A, Perez I, Ruiz-Mateos E, et al. Rate and predictors of progression in elite and viremic HIV-1 controllers. *AIDS*. 2016;30(8):1209-1220. doi:10.1097/QAD.0000000000001050
3. Blankson JN. Control of HIV-1 replication in elite suppressors. *Discov Med*. 2010;9(46):261-266. Accessed October 26, 2020. PMID 20350494
4. Pernas M, Tarancón-Diez L, Rodríguez-Gallego E, et al. Factors Leading to the Loss of Natural Elite Control of HIV-1 Infection. *J Virol*. 2018;92(5). doi:10.1128/jvi.01805-17
5. Noel N, Lerolle N, Lécuroux C, et al. Immunologic and virologic progression in HIV controllers: The role of viral "blips" and immune activation in the ANRS CO21 CODEX study. *PLoS One*. 2015;10(7). doi:10.1371/journal.pone.0131922
6. Boufassa F, Saez-Cirion A, Lechenadec J, et al. CD4 dynamics over a 15 year-period among HIV controllers enrolled in the ANRS French Observatory. *PLoS One*. 2011;6(4). doi:10.1371/journal.pone.0018726
7. Egaña-Gorroño L, Escribà T, Boulanger N, et al. Differential MicroRNA expression profile between stimulated PBMCs from HIV-1 infected elite controllers and viremic progressors. *PLoS One*. 2014;9(9). doi:10.1371/journal.pone.0106360
8. Rotger M, Dang KK, Fellay J, et al. Genome-wide mRNA expression correlates of viral control in CD4+ T-Cells from HIV-1-infected individuals. *PLoS Pathog*. 2010;6(2). doi:10.1371/journal.ppat.1000781
9. Swaminathan G, Martin-Garcia J, Navas-Martin S. RNA viruses and microRNAs: Challenging discoveries for the 21st century. *Physiol Genomics*. 2013;45(22):1035-1048. doi:10.1152/physiolgenomics.00112.2013
10. Houzet L, Yeung ML, de Lame V, Desai D, Smith SM, Jeang KT. MicroRNA profile changes in human immunodeficiency virus type 1 (HIV-1) seropositive individuals. *Retrovirology*. 2008;5. doi:10.1186/1742-4690-5-118
11. Kim VN, Nam JW. Genomics of microRNA. *Trends Genet*. 2006;22(3):165-173. doi:10.1016/j.tig.2006.01.003
12. Ayala-Suárez R, Díez-Fuertes F, Calonge E, et al. Insight in miRNome of Long-Term Non-Progressors and Elite Controllers Exposes Potential RNAi Role in Restraining HIV-1 Infection. *J Clin Med*. 2020;9(8):2452. doi:10.3390/jcm9082452
13. Asahchop EL, Akinwumi SM, Branton WG, Fujiwara E, Gill MJ, Power C. Plasma microRNA profiling predicts HIV-associated neurocognitive disorder. *AIDS*. 2016;30(13):2021-2031. doi:10.1097/QAD.0000000000001160
14. Low H, Hoang A, Pushkarsky T, et al. HIV disease, metabolic dysfunction and atherosclerosis: A three year prospective study. *PLoS One*. 2019;14(4). doi:10.1371/journal.pone.0215620
15. Mallol R, Amigó N, Rodríguez MA, et al. Liposcale: A novel advanced lipoprotein test based on 2D diffusion-ordered 1H NMR spectroscopy. *J Lipid Res*. 2015;56(3):737-746. doi:10.1194/jlr.D050120
16. Chang L, Zhou G, Soufan O, Xia J. miRNet 2.0: network-based visual analytics for miRNA functional analysis and systems biology. *Nucleic Acids Res*. 2020;48(W1):W244-W251. doi:10.1093/nar/gkaa467
17. Tarancon-Diez L, Rodríguez-Gallego E, Rull A, et al. Immunometabolism is a key factor for the persistent spontaneous elite control of HIV-1 infection. *EBioMedicine*. 2019;42:86-96. doi:10.1016/j.ebiom.2019.03.004
18. Zhang L, Chen X, Shi Y, et al. miR-27a suppresses EV71 replication by directly targeting EGFR. *Virus Genes*. 2014;49(3):373-382. doi:10.1007/s11262-014-1114-4

19. Xie N, Cui H, Banerjee S, et al. miR-27a Regulates Inflammatory Response of Macrophages by Targeting IL-10. *J Immunol*. 2014;193(1):327-334. doi:10.4049/jimmunol.1400203
20. Rodríguez-Gallego E, Tarancón-Diez L, Garciá F, et al. Proteomic profile associated with loss of spontaneous human immunodeficiency virus type 1 elite control. *J Infect Dis*. 2019;219(6):867-876. doi:10.1093/infdis/jiy599
21. Palmer CS, Ostrowski M, Gouillou M, et al. Increased glucose metabolic activity is associated with CD4+ T-cell activation and depletion during chronic HIV infection. *AIDS*. 2014;28(3):297-309. doi:10.1097/QAD.0000000000000128
22. Kadri F, Laplante A, De Luca M, et al. Defining Plasma MicroRNAs Associated With Cognitive Impairment In HIV-Infected Patients. *J Cell Physiol*. 2016;231(4):829-836. doi:10.1002/jcp.25131
23. Squillace N, Bresciani E, Torsello A, et al. Changes in subcutaneous adipose tissue microRNA expression in HIV-infected patients. *J Antimicrob Chemother*. 2014;69(11):3067-3075. doi:10.1093/jac/dku264
24. Sevastianova K, Sutinen J, Kannisto K, Hamsten A, Ristola M, Yki-Järvinen H. Adipose tissue inflammation and liver fat in patients with highly active antiretroviral therapy-associated lipodystrophy. *Am J Physiol - Endocrinol Metab*. 2008;295(1). doi:10.1152/ajpendo.90224.2008
25. Kotler DP, Ionescu G, Johnson JA, et al. Studies of adipose tissue metabolism in human immunodeficiency virus - Associated lipodystrophy. *Clin Infect Dis*. 2003;37(SUPPL. 2). doi:10.1086/375891
26. Hariharan M, Scaria V, Pillai B, Brahmachari SK. Targets for human encoded microRNAs in HIV genes. *Biochem Biophys Res Commun*. 2005;337(4):1214-1218. doi:10.1016/j.bbrc.2005.09.183
27. Klase Z, Houzet L, Jeang KT. MicroRNAs and HIV-1: Complex interactions. *J Biol Chem*. 2012;287(49):40884-40890. doi:10.1074/jbc.R112.415448
28. Balasubramaniam M, Pandhare J, Dash C. Are microRNAs important players in HIV-1 infection? An update. *Viruses*. 2018;10(3). doi:10.3390/v10030110
29. Ahluwalia JK, Khan SZ, Soni K, et al. Human cellular microRNA hsa-miR-29a interferes with viral nef protein expression and HIV-1 replication. *Retrovirology*. 2008;5:117. doi:10.1186/1742-4690-5-117
30. Wang R, Li G, Zhuang G, Sun S, Song Z. Overexpression of microRNA-423-3p indicates poor prognosis and promotes cell proliferation, migration, and invasion of lung cancer. *Diagn Pathol*. 2019;14(1). doi:10.1186/s13000-019-0831-3
31. Kumar A, Kumar A, Ingle H, et al. MicroRNA hsa-miR-324-5p Suppresses H5N1 Virus Replication by Targeting the Viral PB1 and Host CUEDC2. *J Virol*. 2018;92(19). doi:10.1128/jvi.01057-18
32. Smith KM, Guerau-de-Arellano M, Costinean S, et al. miR-29ab1 Deficiency Identifies a Negative Feedback Loop Controlling Th1 Bias That Is Dysregulated in Multiple Sclerosis. *J Immunol*. 2012;189(4):1567-1576. doi:10.4049/jimmunol.1103171
33. Swaminathan G, Navas-Martín S, Martín-García J. MicroRNAs and HIV-1 infection: Antiviral activities and beyond. *J Mol Biol*. 2014;426(6):1178-1197. doi:10.1016/j.jmb.2013.12.017
34. Nathans R, Chu C ying, Serquina AK, Lu CC, Cao H, Rana TM. Cellular MicroRNA and P Bodies Modulate Host-HIV-1 Interactions. *Mol Cell*. 2009;34(6):696-709. doi:10.1016/j.molcel.2009.06.003
35. Du C, Liu C, Kang J, et al. MicroRNA miR-326 regulates TH-17 differentiation and is associated with the pathogenesis of multiple sclerosis. *Nat Immunol*. 2009;10(12):1252-1259. doi:10.1038/ni.1798
36. He JF, Luo YM, Wan XH, Jiang D. Biogenesis of MiRNA-195 and its role in biogenesis, the cell cycle, and apoptosis. *J Biochem Mol Toxicol*. 2011;25(6):404-408.

doi:10.1002/jbt.20396

37. Zahoor MA, Yao XD, Henrick BM, et al. Expression profiling of human milk derived exosomal microRNAs and their targets in HIV-1 infected mothers. *Sci Rep*. 2020;10(1):1-16. doi:10.1038/s41598-020-69799-x
38. Guo ST, Jiang CC, Wang GP, et al. MicroRNA-497 targets insulin-like growth factor 1 receptor and has a tumour suppressive role in human colorectal cancer. *Oncogene*. 2013;32(15):1910-1920. doi:10.1038/onc.2012.214
39. Shen CJ, Jia YH, Tian RR, Ding M, Zhang C, Wang JH. Translation of Pur-α is targeted by cellular miRNAs to modulate the differentiation-dependent susceptibility of monocytes to HIV-1 infection. *FASEB J*. 2012;26(11):4755-4764. doi:10.1096/fj.12-209023
40. Chepenik LG, Tretiakova AP, Krachmarov CP, Johnson EM, Khalili K. The single-stranded DNA binding protein, Pur-α, binds HIV-1 TAR RNA and activates HIV-1 transcription. *Gene*. 1998;210(1):37-44. doi:10.1016/S0378-1119(98)00033-X
41. Gallia GL, Darbinian N, Tretiakova A, et al. Association of HIV-1 Tat with the cellular protein, Purα, is mediated by RNA. *Proc Natl Acad Sci U S A*. 1999;96(20):11572-11577. doi:10.1073/pnas.96.20.11572
42. Canfrán-Duque A, Ramírez CM, Goedeke L, Lin CS, Fernández-Hernando C. MicroRNAs and HDL life cycle. *Cardiovasc Res*. 2014;103(3):414-422. doi:10.1093/cvr/cvu140
43. Rayner KJ, Suárez Y, Dávalos A, et al. MiR-33 contributes to the regulation of cholesterol homeostasis. *Science (80-)*. 2010;328(5985):1570-1573. doi:10.1126/science.1189862
44. Aryal B, Singh AK, Rotllan N, Price N, Fernández-Hernando C. MicroRNAs and lipid metabolism. *Curr Opin Lipidol*. 2017;28(3):273-280. doi:10.1097/MOL.0000000000000420
45. Wagschal A, Najafi-Shoushtari SH, Wang L, et al. Genome-wide identification of microRNAs regulating cholesterol and triglyceride homeostasis. *Nat Med*. 2015;21(11):1290-1297. doi:10.1038/nm.3980
46. Goedeke L, Rotllan N, Ramírez CM, et al. miR-27b inhibits LDLR and ABCA1 expression but does not influence plasma and hepatic lipid levels in mice. *Atherosclerosis*. 2015;243(2):499-509. doi:10.1016/j.atherosclerosis.2015.09.033
47. Sun L, Trajkovski M. MiR-27 orchestrates the transcriptional regulation of brown adipogenesis. *Metabolism*. 2014;63(2):272-282. doi:10.1016/j.metabol.2013.10.004
48. Khan AA, Agarwal H, Reddy SS, et al. MicroRNA 27a Is a Key Modulator of Cholesterol Biosynthesis. *Mol Cell Biol*. 2020;40(9). doi:10.1128/mcb.00470-19
49. Jopling CL, Yi MK, Lancaster AM, Lemon SM, Sarnow P. Molecular biology: Modulation of hepatitis C virus RNA abundance by a liver-specific MicroRNA. *Science (80-)*. 2005;309(5740):1577-1581. doi:10.1126/science.1113329
50. Martínez-González E, Brochado-Kith Ó, Gómez-Sanz A, et al. Comparison of methods and characterization of small RNAs from plasma extracellular vesicles of HIV/HCV coinfected patients. *Sci Rep*. 2020;10(1). doi:10.1038/s41598-020-67935-1
51. Zhang J, Jazii FR, Haghighi MM, et al. MiR-130b is a potent stimulator of hepatic very-low-density lipoprotein assembly and secretion via marked induction of microsomal triglyceride transfer protein. *Am J Physiol - Endocrinol Metab*. 2020;318(2):E262-E275. doi:10.1152/ajpendo.00276.2019

Annex S1: Clinical Centres and research groups which contribute to ECRIS:

Clinical centres:

Hospital Universitario de Valme (Sevilla): Juan Antonio Pineda, Pilar Rincón, Juan Macías Sanchez, Luis Miguel Real, Anais Corma Gomez, Marta Fernandez Fuertes, Alejandro Gonzalez-Serna.

Hospital General Universitario Santa Lucía (Cartagena): Onofre Juan Martínez, Lorena Martinez, Francisco Jesús Vera, Josefina García, Begoña Alcaraz, Amaya Jimeno.

Hospital Clinic de Barcelona (Barcelona): José M. Miró, Christian Manzardo, Laura Zamora, Iñaki Pérez, Mª Teresa García, Carmen Ligero, José Luis Blanco, Felipe García-Alcaide, Esteban Martínez, Josep Mallolas, José M. Gatell.

Hospital General Universitario de Alicante (Alicante): Joaquín Portilla, Irene Portilla, Esperanza Merino, Gema García, Iván Agea, José Sánchez-Payá, Juan Carlos Rodríguez, Lina Gimeno, Livia Giner, Melissa Carreres, Sergio Reus, Vicente Boix, Diego Torrus, Verónica Pérez Esquerdo, Julia Portilla Tamarit.

Hospital Universitari de Bellvitge (Hospitalet de Llobregat): Daniel Podzamczer, Arkaitz Imaz, Juan Tiraboschi, Ana Silva, María Saumoy, Paula Prieto, Sofía Scevola.

Hospital Universitario de Canarias (Santa Cruz de Tenerife): Juan Luís Gómez Sirvent, Jehovana Hernández, Ana López Lirola, Dácil García, Felicitas Díaz-Flores, María del Mar Alonso, Ricardo Pelazas, María Remedios Alemán.

Hospital Carlos III (Madrid): Vicente Soriano, Pablo Labarga, Pablo Barreiro, Pablo Rivas, Francisco Blanco, Luz Martín Carbonero, Eugenia Vispo, Carmen Solera.

Hospital Universitario Central de Asturias (Oviedo): Victor Asensi, María Eugenia Rivas Carmenado, Tomas Suarez-Zarracina Secades.

Hospital Doce de Octubre (Madrid): Federico Pulido, Rafael Rubio, Otilia Bisbal, Mª Asunción Hernando, David Rial Crestelo, María de Lagarde, Rebeca Font, Octavio Arce, Adriana Pinto, Laura Bermejo, Mireia Santacreu.

Hospital Universitario Donostia (San Sebastián): Jose Antonio Iribarren, MªJosé Aramburu, Julio Arrizabalaga, Xabier Camino, Francisco Rodríguez-Arrondo, Miguel Ángel von Wichmann, Lidia Pascual Tomé, Miguel Ángel Goenaga, Mª Jesús Bustinduy, Harkaitz Azkune, Maialen Ibarguren, Xabier Kortajarena, Mª Pilar Carmona Oyaga, Ainhoa Garaialde Fernandez.

Hospital General Universitario de Elche (Elche): Félix Gutiérrez, Catalina Robledano, Mar Masiá, Sergio Padilla, Araceli Adsuar, Rafael Pascual, Marta Fernández, Antonio Galiana, José Alberto García, Xavier Barber, Vanessa Agullo Re, Javier Garcia Abellan, Reyes Pascual Pérez, Guillermo Telenti , Lucia Guillén, Angela Botella.

Hospital Germans Trías i Pujol (Badalona): Roberto Muga, Arantza Sanvisens, Daniel Fuster.

Hospital General Universitario Gregorio Marañón (Madrid): Juan Berenguer, Isabel Gutierrez Cuellar, Juan Carlos López Bernaldo de Quirós, Margarita Ramírez, Belén Padilla, Paloma Gijón, Teresa Aldamiz-Echevarría, Francisco Tejerina, Cristina Diez, Leire Pérez Latorre, Chiara Fanciulli, Saray Corral Carretero.

Hospital Universitari de Tarragona Joan XXIII, IISPV, Universitat Rovira i Virgili (Tarragona): Francesc Vidal, Anna Marti, Joaquín Peraire, Consuelo Viladés, Sergio Veloso, Montserrat Vargas, Montserrat Olona, Anna Rull, Verónica Alba, Miguel López-Dupla, Elena Yeregui, Jenifer Masip, Laia Reverté.

Hospital Universitario La Fe (Valencia): Marta Montero Alonso, Sandra Cuéllar Tovar, Marino Blanes Juliá, María Tasias Pitarch, Eva Calabuig Muñoz , Miguel Salavert Lletí , Juan Fernández Navarro.

Hospital Universitario La Paz/IdiPaz (Madrid): Juan González-Garcia, Ana Delgado Hierro, Francisco Arnalich, José Ramón Arribas, Jose Ignacio Bernardino de la Serna, Juan Miguel Castro, Luis Escosa, Pedro Herranz, Victor Hontañón, Silvia García-Bujalance, Milagros García López-Hortelano, Alicia González-Baeza, Maria Luz Martín-Carbonero, Mario Mayoral, Maria Jose Mellado, Rafael Esteban Micán, Rocio Montejano, María Luisa Montes, Victoria Moreno, Ignacio Pérez-Valero, Berta Rodés, Guadalupe Rúa Cebrián, Talia Sainz, Elena Sendagorta, Eulalia Valencia, Carmen Busca, Joanna Cano, Julen Cardiñanos, Rosa de Miguel.

Hospital de la Princesa (Madrid): Ignacio de los Santos Gil, Alejandro de los Santos San Frutos, Jesús Sanz Sanz, Lucio Garcia-Fraile Fraile, Enrique Martín Gayo, Ildefonso Sánchez-Cerrillo, Marta Calvet i Mirabent.

Hospital San Pedro-CIBIR (Logroño): José Antonio Oteo, José Ramón Blanco, Valvanera Ibarra, Luis Metola, Mercedes Sanz, Laura Pérez-Martínez.

Complejo Hospitalario de Navarra (Pamplona): María Rivero, Beatriz Piérola Ruiz de Galarreta, Maider Goikoetxea Peñagarikano, María Gracia Ruiz de Alda, Carlos Ibero Esparza, Estela Moreno García, Jesús Repáraz.

Hospital Parc Taulí (Sabadell): Gemma Navarro, María José Amengual, Manel Cervantes Garcia, Sonia Calzado Isbert, Marta Navarro Vilasaro, Belen Lopez Garcia.

Hospital Ramón y Cajal (Madrid): Santiago Moreno, Santos del Campo, José Luis Casado Osorio, Fernando Dronda Nuñez, Ana Moreno Zamora, Maria Jesús Pérez Elías, Carolina Gutiérrez, Nadia Madrid, Sergio Serrano Villar, Maria Jesús Vivancos Gallego, Javier Martínez Sanz, Tamara Velasco, Alejandro Vallejo, Matilde Sanchez Conde, Jose Antonio Pérez Molina.

Hospital Reina Sofía (Murcia): Enrique Bernal, Antonia Alcaraz, Joaquín Bravo Urbieta, Angeles Muñoz Perez, Cristina Tomás Jimenez, Monica Martinez Martinez, Maria Jose Alcaraz, Maria del Carmen Villalba.

Hospital San Cecilio (Granada): Federico García, Clara Martínez, José Hernández Quero, Leopoldo Muñoz Medina, Marta Alvarez, Natalia Chueca, David Vinuesa García, Adolfo de Salazar Gonzalerz, Ana Fuentes Lopez.

Centro Sanitario Sandoval (Madrid): Jorge Del Romero Guerrero, Montserrat Raposo Utrilla, Carmen Rodríguez, Teresa Puerta, Juan Carlos Carrió, Mar Vera, Juan Ballesteros, Oskar Ayerdi.

Hospital Son Espases (Palma de Mallorca): Melchor Riera, María Peñaranda, Mª Angels Ribas, Antonia Campins, Carmen Vidal, Francisco Fanjul, Javier Murillas, Francisco Homar, Helem H Vilchez, Maria Luisa Martin, Antoni Payeras.

Hospital Universitario Virgen del Rocío (Sevilla): Luis Fernando López-Cortés, Silvia Llaves, Nuria Espinosa.

Research groups:

Hospital General Universitario Gregorio Marañón e Insituto de Investigación Sanitaria Gregorio Marañón. Maria Angeles Muñoz-Fernández, Laura Tarancon-Diez, Jose Luis Jimenez, Daniel Sepúlveda, Rafael Ceña, Isabel García Merino, Irene Consuegra.

Hospital Clinic. Agathe León, Montse Plana, Nuria Climent, Felipe García.

Hospital Joan XXIII. Francesc Vidal, Anna Marti, Joaquín Peraire, Consuelo Viladés, Sergio Veloso, Montserrat Vargas, Montserrat Olona, Anna Rull, Verónica Alba, Miguel López-Dupla, Elena Yeregui, Jenifer Masip, Laia Reverté.

IIS-Fundacion Jimenez Díaz, UAM. Jose Miguel Benito, Norma Rallón, Clara Restrepo, Marcial García, Alfonso Cabello, Miguel Gorgolas.

Centro Sandoval. Jorge Del Romero, Carmen Rodríguez, Mar Vera.

Fundacion IRSI CAIXA. José Esté, Esther Ballana, Miguel Angel Martinez, S Franco, María Nevot, Julia G. Prado, Esther Jiménez

Hospital Ramón y Cajal. Alejandro Vallejo, Beatriz Sara Sastre, Santiago Moreno.

Virologia Molecular ISCIII. Maria Pernas, Concepción Casado, Cecilio López Galíndez

Infeccion viral e Inmunidad. ISCIII. Salvador Resino

Inmunopatología del SIDA. ISCIII. Laura Capa, Mayte Perez-Olmeda, Pepe Alcami

Mutacion y evolución de virus. Univ Valencia. Rafael Sanjuán, José Manuel Cuevas

Hospital Universitario Doce de Octubre (Madrid): Rafael Rubio, Federico Pulido, Otilia Bisbal, Mª Asunción Hernando, Mariano Matarranz, María Lagarde, Lourdes Domínguez.

Universidad de la Laguna. Agustín Valenzuela-Fernández.

Hospital Virgen del Rocio: Ezequiel Ruiz-Mateos, María Reyes Jiménez-León, Carmen Gasca-Capote, Alberto Pérez-Gómez, Mohamed Rafii-El-Idrissi Benhnia, Alicia Gutierrez-Valencia, María Trujillo, Ana Serna-Gallego, Esperanza Muñoz-Muela, Silvia Llaves, Cristina Roca-Oporto, Nuria Espinosa, Luis Fernando López-Cortés
